# Exogenous and endogenous antioxidants in osteoporosis risk: causal associations unveiled by Mendelian Randomization analysis

**DOI:** 10.3389/fphys.2024.1411148

**Published:** 2024-05-31

**Authors:** Yuancheng Li, Huaqian Qi, Xin Huang, Gang Lu, Huashan Pan

**Affiliations:** ^1^ Clinical Medical College of Acupuncture-Moxibustion and Rehabilitation, Guangzhou University of Chinese Medicine, Guangzhou, China; ^2^ School of Physical Education and Health, Guangzhou University of Chinese Medicine, Guangzhou, China; ^3^ Department of Acupuncture-Moxibustion and Tuina, Shaanxi University of Chinese Medicine, Xianyang, China

**Keywords:** oxidative stress, endogenous antioxidant, exogenous antioxidant, osteoporosis, albumin, Mendelian Randomization analysis

## Abstract

**Background:**

Recent epidemiological studies and animal experiments have highlighted the significant role of oxidative stress in the development of osteoporosis (OP). The provision of antioxidants is widely considered a fundamental strategy to combat free radical-induced stress, inhibit oxidative damage, and potentially reverse the adverse effects of oxidative stress on bone health. However, there is no consensus in the scientific literature regarding the practical effectiveness of antioxidants in OP prevention and treatment. Some studies have not shown a clear connection between antioxidant supplementation and decreased OP risk. Therefore, it is essential to clarify the potential causal relationship between antioxidants and the development of OP.

**Methods:**

The study utilized the inverse variance weighted (IVW) approach as the primary analytical method in the Mendelian Randomization (MR) framework to investigate the causal effects of five exogenous and six endogenous antioxidants on the risk of OP. To thoroughly assess potential pleiotropic effects and heterogeneity among the data analyzed, the MR-Egger intercept test was employed, and Cochran’s Q statistic was calculated.

**Results:**

In the evaluation of exogenous antioxidants, single-directional two-sample MR analyses did not reveal any statistically significant relationship between these agents and the risk of OP. Regarding endogenous antioxidants, bidirectional two-sample MR analyses were conducted, which generally indicated that most genetically regulated endogenous antioxidants had no significant association with the onset risk of OP. A significant causal relationship was found between OP and serum albumin levels (*β*: −0.0552, 95%CI: −0.0879 to −0.0225, *p* < 0.0011 after Bonferroni adjustment, power = 100%).

**Conclusion:**

The research uncovers OP as a possible determinant contributing to a decrement in serum albumin levels, and further suggests a potentially intimate relationship between the downward trajectory of serum albumin concentrations and the advancement of the OP disease process.

## 1 Introduction

In 1993, the World Health Organization (WHO) classified osteoporosis (OP) as a systemic skeletal disease. OP is characterized by low bone density, deterioration of bone tissue microstructure, increased bone fragility, and susceptibility to fracture (Genant et al., 1999). As a severe, chronic, progressive, and asymptomatic metabolic bone disease, OP is a major public health concern worldwide (LeBoff et al., 2022; Moore et al., 2023). The statistical evidence indicates that over 200 million people worldwide suffer from OP. Women over the age of 50 are particularly affected by this condition, with almost one-third being affected. Additionally, about one-fifth of men experience fractures related to osteoporosis during their lifetime (Sozen et al., 2017; Gregson et al., 2022).

In recent years, OP has become a pressing emerging health issue that requires prompt attention. It imposes considerable economic and caregiving burdens on societal healthcare resources and individual households (Mottaghi and Nasri, 2021). The pathophysiology of OP involves a dysregulation in bone metabolism, characterized by an excessive rate of bone resorption over bone formation. This, in turn, results in reduced bone mass and declining bone mineral density (BMD) (Liang et al., 2022). Historically, estrogen deficiency has been identified as a key trigger for OP. However, recent epidemiological and experimental animal studies have highlighted the significant role of oxidative stress in the OP disease process. This underscores the close relationship between bone density and the oxidative state within the organism (Manolagas, 2010; Zhang et al., 2023).

Normally, intracellular reactive oxygen species (ROS) are effectively scavenged by the body’s antioxidant systems to maintain cellular redox homeostasis (de Almeida et al., 2022). ROS act as dual-effect molecules, serving as crucial secondary messengers involved in regulating essential life processes such as apoptosis, differentiation, and proliferation at appropriate concentrations. This has significant implications for the dynamic balance between osteoblasts and osteoclasts (Zhu et al., 2022; Endale et al., 2023). However, studies have shown that aging, inflammatory responses and estrogen deficiency can increase the production of ROS. When the rate of ROS generation exceeds the body’s intrinsic antioxidant clearance capacity, it results in a state of oxidative stress (Mohamad et al., 2020; Zhu et al., 2022; Iantomasi et al., 2023) Excessive accumulation of ROS can suppress osteoblast differentiation and proliferation, while promoting osteoclast differentiation. This can cause structural disruption of bone tissue and subsequent loss of bone mass (Kimball et al., 2021). Additionally, it is noteworthy that the decline in estrogen levels often coincides with elevated levels of pro-inflammatory factors. This further exacerbates ROS production and consequently elevates the risk of OP (Zhou et al., 2021).

**FIGURE 1:**
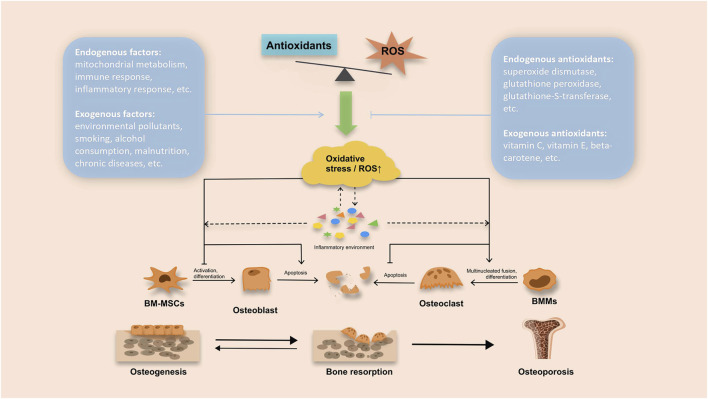
Oxidative stress and antioxidant mechanisms in the development of osteoporosis.

Research has shown that supplementing with antioxidants is a key approach to reducing the impact of free radicals, preventing oxidative damage, and countering the negative effects of oxidative stress on bone health (Damani et al., 2022; Davan et al., 2023). Figure 1 illustrates the oxidative stress and antioxidant mechanisms involved in the development of OP. Dietary exogenous antioxidants mainly comprise carotenoids, retinol, vitamin C, and vitamin E. On the other hand, endogenous constituents primarily consist of albumin, bilirubin, and key antioxidant enzymes such as Catalase (CAT), Superoxide Dismutase (SOD), Glutathione Peroxidase (GPX), and Glutathione S-transferase (GST) (Behera and Malik, 1989; Jomová et al., 2023). Research has shown that osteoporotic rats have notably depleted serum glutathione levels (Yalin et al., 2012; Ameen et al., 2020). Furthermore, increasing the GSH/GSSG ratio can help mitigate ROS-induced oxidative injury to osteoblasts by activating the PI3K/Akt-Nrf2 signaling cascade (Casati et al., 2019). However, when examining the relationship between antioxidant biomarkers and OP, conflicting results have been reported. One systematic appraisal found lower SOD activity in postmenopausal women with OP (Zhou et al., 2016), while a case-control investigation showed significantly higher plasma SOD activity in osteoporotic cases compared to controls (Malekian et al., 2023). The effects of dietary-exogenous antioxidants and their metabolites on OP also remain inconsistent. Numerous studies have demonstrated that exogenous antioxidants, including carotenoids, vitamin C, and vitamin E, can reduce the risk of OP (Chavan et al., 2007; Yang et al., 2008; Xu et al., 2016). In studies investigating exogenous antioxidant metabolites, Maggio observed plasma antioxidant levels in patients with osteoporosis, noting consistently lower mean plasma levels of vitamin C, vitamin E, and vitamin A in osteoporosis patients compared to the control group (Maggio et al., 2003). Additionally, Zhang et al. observed a U-shaped correlation between plasma retinol concentration and BMD, suggesting that an appropriate plasma retinol concentration may contribute to improving bone mineral status (Zhang et al., 2022). However, some studies have yielded conflicting conclusions. For instance, Barker et al.’s study failed to find a significant correlation between serum *β*-carotene levels and the risk of hip fractures in elderly women, indicating a lack of association between *β*-carotene and reduced OP or fracture risk (Barker et al., 2005). Furthermore, a substudy of the Women’s Health Initiative (WHI) examined the associations between serum levels of *α*-tocopherol and *γ*-tocopherol, dietary and total vitamin E intake, and BMD in postmenopausal women. It found no significant correlation between dietary or total vitamin E intake, serum tocopherol concentrations, and BMD (Wolf et al., 2005). Consequently, the scientific community has not yet reached a consensus on the precise role of antioxidants in the prevention and treatment of osteoporosis.

Observational studies often face complexities in discerning a direct causal relationship between antioxidants and OP due to confounding factors, potential reverse causality, limitations imposed by sample size, and measurement errors (Sekula et al., 2016). To overcome these issues, Mendelian Randomization (MR) research emerges as an ideal methodology due to its unique theoretical framework and practical advantages. This technique uses naturally occurring genetic Instrumental Variables (IVs) to indirectly infer causality between genetically determined biological exposures and health outcomes. In this study, we will use external or exogenous antioxidants and related metabolites as the exposure variable and OP as the outcome variable. Using a unidirectional two-sample MR design, we investigated whether there is a causal link between exogenous antioxidants and OP incidence. Additionally, employing a bidirectional two-sample MR approach, we assessed the relationship between endogenous antioxidants and the risk of OP. These analyses aim to provide insights into the causal relationships between antioxidants and OP, contributing to the understanding of their roles in the pathogenesis and potential prevention strategies for OP.

## 2 Materials and methods

### 2.1 Research design

**FIGURE 2:**
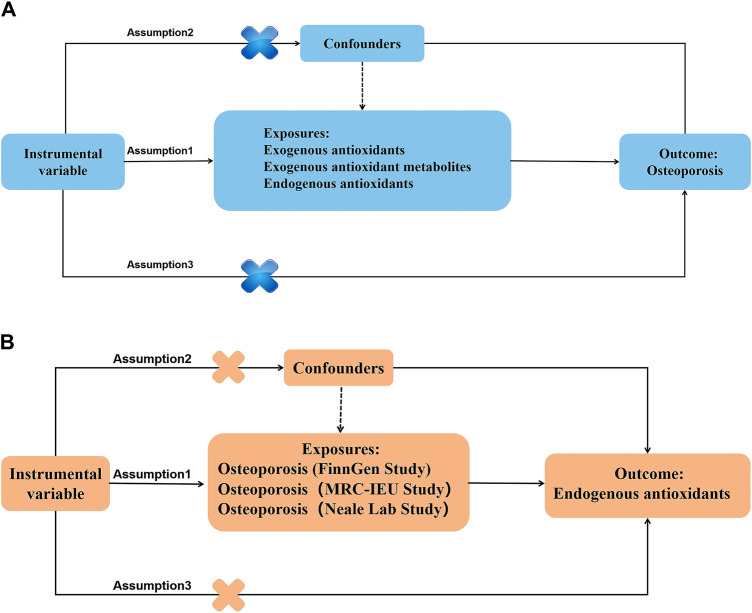
Main design of the study. **(A)** The MR hypothesis diagram of antioxidants on osteoporosis. **(B)** The MR hypothesis diagram of osteoporosis on endogenous antioxidants.

**FIGURE 3:**
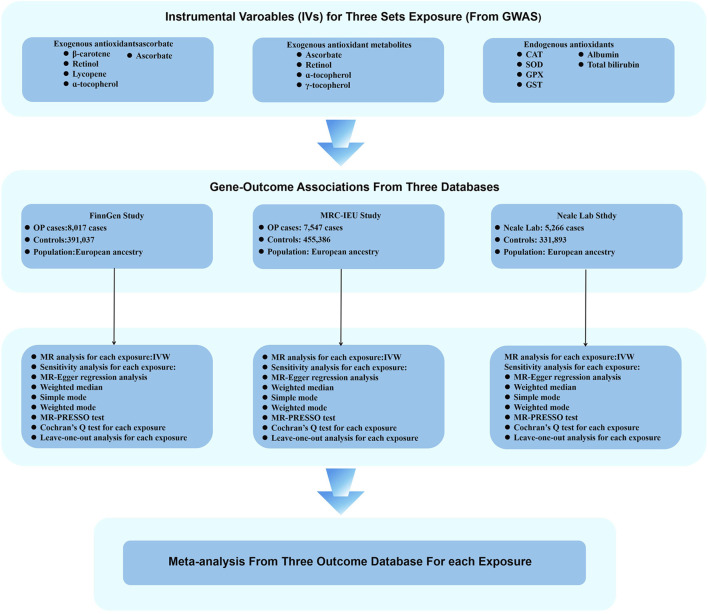
Flow chart illustrating the MR study design investigating the relationship between antioxidants and OP. During the exposure variable identification phase, SNPs for exogenous antioxidants, exogenous antioxidant metabolites, and endogenous antioxidants were used as IVs. In the analytical phase of assessing associations between genes and outcomes, three databases were employed as outcome variables: the FinnGen study, the MRC-IEU study, and the Neale Laboratory study. Ultimately, a meta-analysis of the MR analysis results led to the inference of a causal relationship between antioxidants and OP.

**FIGURE 4:**
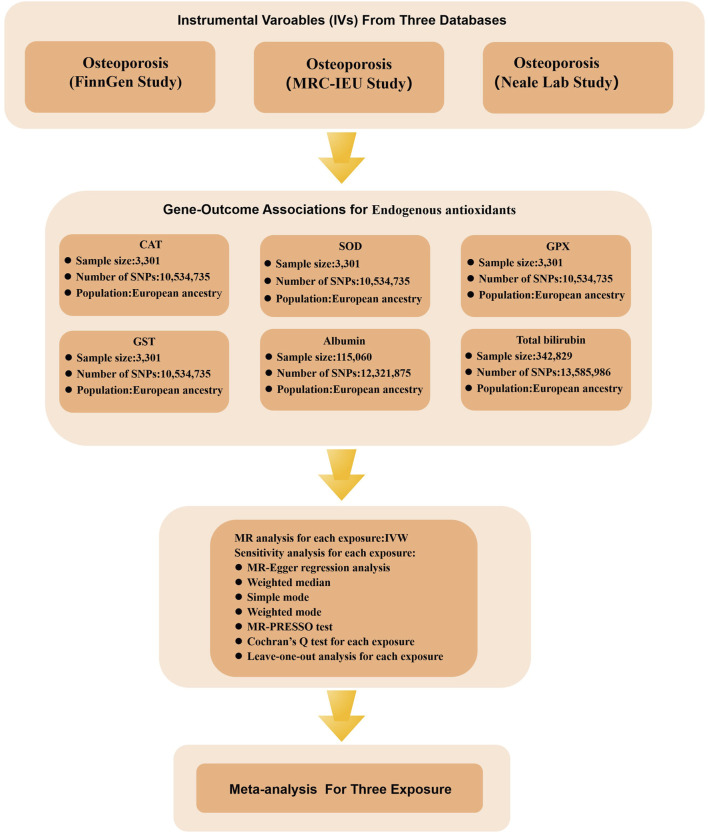
Flow chart illustrating the MR study design investigating the relationship between OP and endogenous antioxidants. During the exposure variable identification phase, data from three distinct sources of OP were utilized: the FinnGen Study, the MRC-IEU Study, and the Neale Lab Study. In the gene-outcome association analysis phase, six different endogenous antioxidants were employed as outcome variables. Ultimately, a meta-analysis of the MR analysis results led to the inference of a causal relationship between OP and endogenous antioxidants.

Figures 2–4 provide a schematic overview of the MR design used in this study. A unidirectional two-sample MR approach was employed to evaluate the potential causal association between absolute circulating levels of exogenous antioxidants and their corresponding metabolites and the risk of OP. Additionally, a bidirectional two-sample MR strategy was utilized to investigate the causal relationship between endogenous antioxidants and OP incidence. The analysis of the MR was based on three fundamental assumptions. Firstly, the genetic variants used should be associated with the exposure. Secondly, the outcome should only be influenced by the exposure. Finally, the genetic variants should be independent of any measured or unmeasured confounders. The data used in this study were obtained from publicly available sources and were accompanied by ethical approvals and informed consent in each of the original studies from which they were derived (Richmond and Davey Smith, 2021).

### 2.2 Selection of genetic instruments

This study focuses on five central dietary antioxidants and six endogenous antioxidants. The concentrations of exogenous antioxidants in blood and their related metabolites were evaluated, and their concentrations in plasma or serum were quantified using relative units. The study employed various exogenous and endogenous antioxidants, including ascorbate, *β*-carotene, lycopene, *α*-tocopherol, *γ*-tocopherol, retinol, CAT, SOD, GPX, GST, albumin, and total Bilirubin. The Genome-wide Association Studies (GWAS) data for OP were obtained from the GWAS database (http://gwas-api.mrcieu.ac.uk/) and the FinnGen consortium (https://www.finngen.fi/en).

To obtain IVs that satisfy the three critical assumptions necessary for robust and reliable MR analysis, we implemented a series of stringent quality control measures:

• Firstly, we identified genome-wide significant SNPs independently associated with each exposure, ensuring a direct genetic influence on the studied trait without residual confounding.
• Secondly, we eliminated linkage disequilibrium among SNPs to avoid inflated statistical associations due to non-independent genetic variants.
• Thirdly, we utilized the “get_f” functionality provided by the Get_MR software toolkit (https://github.com/HaobinZhou/Get_MR). This tool assists in computing both the squared correlation coefficient (*R* ^2^) and the F-statistic, enabling us to discard weak instrumental variables. Specifically, we retained SNPs with F-statistics greater than 10 to strengthen the reliability of our genetic instruments.
• Fourthly, to ensure accurate results and mitigate issues related to ambiguous allele phasing, we also removed palindromic variants that exhibited intermediate allele frequencies.

These measures enhance the validity of our MR approach by ensuring that the selected SNPs robustly represent genetic proxies for antioxidant exposure, thereby minimizing the potential for biased or spurious results.

### 2.3 Data sources and SNPs selection for exogenous antioxidant biomarkers

This study extracted ten distinct SNPs related to the metabolism or biological action of ascorbate (vitamin C) from a contemporary GWAS (Zheng et al., 2020). To explore the genetic basis of *β*-carotene levels, we referred to a GWAS involving 2,344 participants enrolled in the Nurses’ Health Study, which yielded three genetic variants significantly associated (*p* $<\;5\times 10^{-8}$ ) and demonstrating low linkage disequilibrium (LD < 0.2) (Hendrickson et al., 2012). In a GWAS of 441 Amish adults, we verified five SNPs linked to serum lycopene concentrations. All exhibited significant associations (*p* $<\;5\times 10^{-6}$ ) and stringent LD thresholds (LD < 0.001) (D’Adamo et al., 2016). Furthermore, we retrieved three SNPs correlated with *α*-tocopherol concentrations from a GWAS study on 4,014 males of European descent that met the same stringency criteria (*p* $<\;5\times 10^{-8}$ , LD < 0.001) (Major et al., 2011). Finally, in a GWAS that pooled data from two cohorts comprising a total of 5,006 Caucasian individuals, we identified two genetic variants that were significantly associated with retinol concentrations (*p* $<5\times 10^{-8}$ , LD < 0.001) (Mondul et al., 2011).

### 2.4 Data sources and SNPs selection for exogenous antioxidant metabolite biomarkers

Exogenous antioxidant metabolites are products formed through the metabolic processing of exogenous antioxidants within the body. Exogenous antioxidants, such as vitamins C and E, carotenoids, and lycopene, which are ingested through the diet, are transformed into various metabolites through digestion and metabolic actions. These metabolites exert antioxidant effects within the body, helping to neutralize free radicals, reduce oxidative stress, and thereby protect cells from damage (Moussa et al., 2019). In a meticulous review of peer-reviewed GWAS data, we identified a set of genetic variants displaying genomewide significance (*p* $<\;1\times 10^{-5}$ ) and associating with a variety of antioxidant metabolite levels. More precisely, we extracted the following genetic instruments about the metabolic traits under scrutiny from two independent studies aggregating 7,824 European adults: (1) Eleven genetic IVs were compiled for *α*-tocopherol concentrations, each hailing from a subsample of 7,276 individuals within the analyzed datasets. (2) A cluster of 13 genetic variants served as IVs for *γ*-tocopherol concentrations, all stemming from a research cohort of 5,822 individuals. (3) Regarding plasma ascorbate (L-ascorbate) concentrations, we pinpointed 14 genetic IVs, all sourced from a segment of the referenced studies involving 2,063 subjects (Shin et al., 2014). (4) For retinol metabolism, we unearthed 24 significantly associated genetic IVs in an independent cohort of 1,960 individuals of European ancestry (Long et al., 2017). Considering that *β*-carotene can be converted to retinol in the body, and given the current lack of large-scale GWAS data on metabolites of lycopene, we opted to focus solely on the four exogenous antioxidants for which data were available for further investigation.

### 2.5 Data sources and SNPs selection for endogenous antioxidant biomarkers

Six genetic predictors of endogenous antioxidants were extracted from recent state-of-the-art global genomic studies. The INTERVAL project performed a comprehensive quantitative assessment of 3,622 plasma proteins in 3,301 healthy participants, revealing 27 genetic variants associated with CAT, 23 with SOD, 22 with GPX, and 14 with GST expression (*p* $<\;1\times 10^{-5}$ , LD < 0.001) (Sun et al., 2018). The 30 genetic determinants associated with albumin were from the KORA study (*p* $<\;5\times 10^{-8}$ , LD < 0.001) which included 115,060 individuals (Shin et al., 2014). Eighty-five genetic data related to total bilirubin levels were obtained from an extensive UK biobank that included 342,829 participants (*p* $<\;5\times 10^{-8}$ , LD < 0.001) (Sudlow et al., 2015).

### 2.6 Data sources and SNPs selection for osteoporosis

The OP dataset was compiled by the MRC-IEU using data from the UK Biobank (Sudlow et al., 2015). It comprises 462,933 European individuals, of which 7,547 are cases and 455,386 are controls and covers 9,851,867 SNPs (ukb-b-12141). Additionally, OP-associated SNPs were obtained from the integrated dataset of the Neale Lab Consortium, which included 337,159 Europeans, of which 5,266 were cases and 331,893 were controls and covered 10,894,596 SNPs (ukb-a-87). In addition, the OP dataset of the FinnGen Consortium consists of 399,054 Europeans, including 8,017 cases and 391,037 controls. The dataset contains 19,173,961 SNPs(The dataset is publicly available at the Google Cloud Storage location gs://finngen-public-data-r10/summary_stats/finngen_R10_M13_OSTEOPOROSIS.gz).

### 2.7 Statistical analysis

Statistical analyses were conducted using R version 4.3.1 and software packages “TwoSampleMR,” “MRPRESSO,” and “meta.” The Inverse Variance Weighted (IVW) approach was used as the primary analytical method for MR to investigate the causal relationship between genetic variants associated with exposures and the outcome (Boehm and Zhou, 2022). To guarantee the robustness and dependability of the MR findings, we validated the consistency of these associations using supplementary methods, including MR-Egger, Weighted Median, Simple Mode, and Weighted Mode techniques (Boehm and Zhou, 2022). We evaluated heterogeneity using Cochran’s Q statistic, with a *p*-value < 0.05 indicating heterogeneity and requiring the adoption of a Random Effects IVW method. Otherwise, we used the Fixed Effects IVW method (Bowden et al., 2018). The study assessed the horizontal pleiotropy of SNPs acting as IVs using the MR-Egger intercept test. A *p*-value less than 0.05 suggested the presence of pleiotropy. Additionally, MR-PRESSO was used to detect and correct for overall pleiotropy in the IVs. This resulted in adjusted causal estimates after identifying and excluding outlier instruments (Verbanck et al., 2018).

The study presented the change in natural log-transformed concentrations of *β*-carotene and retinol (using *μ*g/dL and *μ*mol/L as units respectively), *α*-tocopherol (measured in mg/L), lycopene (expressed in *μ*g/dL), and ascorbate (represented in *μ*mol/L) about the risk of OP. The absolute levels of exogenous antioxidants or their corresponding metabolite concentrations when increased tenfold were also considered. In the bidirectional analysis that examined the relationship between endogenous antioxidants and OP, we quantified the effect of a one-standard-deviation (SD) increment in the genetically predicted changes of endogenous antioxidants on the risk of OP as OR. We also assessed the potential influence of OP on the concentrations of six specific endogenous antioxidants, which were conveyed by the *β* coefficients.

In this study, we conducted separate MR analyses using exogenous and endogenous antioxidants as exposure factors and OP data from the MRC-IEU, Neale Lab, and FinnGen datasets as outcome variables, and subsequently meta-analysed the results of the individual studies to obtain a composite estimate of the risk of OP associated with each type of antioxidant exposure. Inversely, we also performed the corresponding MR analyses and meta-analyses using OP as an exposure factor from these three dataset sources with endogenous antioxidants as the outcome variable to derive an estimate of the possible pooled effect of OP status on endogenous antioxidant levels. During the process, we quantified the extent of heterogeneity among the estimates across different studies by computing the I^2^ statistic and using Cochran’s Q test to assess its significance. Depending on the results of heterogeneity testing, we used either a fixed-effect model for meta-analysis in the absence of significant heterogeneity or switched to a random-effects model when substantial heterogeneity was observed.

To mitigate the occurrence of spurious statistically significant results during multiple testing, commonly known as Type I errors, we adopted the Bonferroni correction method for multiple comparisons (VanderWeele and Mathur, 2018). Following this methodological principle, we set the significance threshold at 0.0011 (0.05/45) to determine the presence of an association between the examined variables. Results with a *p*-value below this threshold are considered robust and compelling evidence supporting the presence of an association. Conversely, if the *p*-value falls within the range of 0.0011–0.05, it is considered preliminary indicative evidence suggesting a potential causal relationship.

To ensure that our MR study had sufficient statistical power to detect the anticipated causal effect, we utilized the online MR Power Calculator developed by Simon Briscoe. When the outcome variable is continuous, Eq. 1 is used for the calculation:

$$\text{Power}\approx \Phi \left(\frac{N\cdot R^{2}\cdot \beta^{2}}{\sigma^{2}}-z_{1-\alpha /2}\right)$$ (1)

Equation 1 describes the statistical power calculation used in our study, where Φ is the cumulative distribution function of the standard normal distribution, *N* is the sample size, *R* ^2^ is the proportion of variance explained by the genetic instruments, *β* is the estimated effect size, *σ* ^2^ is the variance of the error term, and *z*_1−*α*/2_ is the critical value from the normal distribution corresponding to a significance level *α*.

## 3 Results

### 3.1 Screening of genetic instruments

Table 1 provides a summary of the genetic instrumental attributes for exogenous and endogenous antioxidants about OP risk. All selected genetic IVs in this investigation exhibited F-statistics surpassing the threshold of 10, indicating their robustness as surrogates for antioxidant exposures. These strong instruments effectively minimized bias in the IVs estimation, thereby enabling more credible causal inferences.

**TABLE 1:** Comprehensive information on studies and associated datasets utilized in the present investigation.

Exposures or outcomes	Trait	Numbers of SNPs	*p*-value	Unit	**R** ^2^	F-statistics	Participants (cases/controls)	PMID
Exogenous antioxidants	*α*-tocopherol	3	5 × 10^−8^	mg/L in log-transformed scale	0.017	86.54	4,014	21,729,881
	Ascorbate	10	5 × 10^−8^	*μ*mol/L	0.017	89.28	52,018	33,203,707
	Lycopene	5	5 × 10^−8^	*μ*g/dL	0.301	189.04	441	26,861,389
	*β*-carotene	2	5 × 10^−8^	*μ*g/L in ln-transformed scale	0.09	231.63	2,344	23,134,893
	Retinol	2	5 × 10^−8^	*μ*g/L in ln-transformed scale	0.023	117.8	5,006	21,878,437
Exogenous antioxidant metabolites	*α*-tocopherol	11	1 × 10^−5^	log10-transformed metabolites concentration	0.033	198.61	7,276	24,816,252
	*γ*-tocopherol	13	1 × 10^−5^	log10-transformed metabolites concentration	0.15	1027.06	5,822	24,816,252
	Retinol	24	1 × 10^−5^	log10-transformed metabolites concentration	0.048	98.57	1,957	28,263,315
	Ascorbate	14	1 × 10^−5^	log10-transformed metabolites concentration	0.186	470.94	2,063	24,816,252
Endogenous antioxidants	CAT	27	1 × 10^−5^	-	0.007	45.89	3,301	29,875,488
	SOD	23	1 × 10^−5^	-	0.008	24.89	3,301	29,875,488
	GPX	22	1 × 10^−5^	-	0.011	25.14	3,301	29,875,488
	GST	14	1 × 10^−5^	-	0.013	38.15	3,301	29,875,488
	Albumin	30	5 × 10^−8^	-	0.0008	90.53	115,060	-
	Total bilirubin	85	5 × 10^−8^	-	0.0006	192.15	342,829	-
Osteoporosis	FinnGen	7	5 × 10^−8^	-	0.00008	32.44	8,017/391,037	-
	MRC-IEU	16	5 × 10^−8^	-	0.0001	45.05	7,547/455,368	-
	Neale Lab	8	5 × 10^−8^	-	0.0001	42.15	5,266/331,893	-

### 3.2 Causal impacts of exogenous antioxidants on osteoporosis risk

**FIGURE 5:**
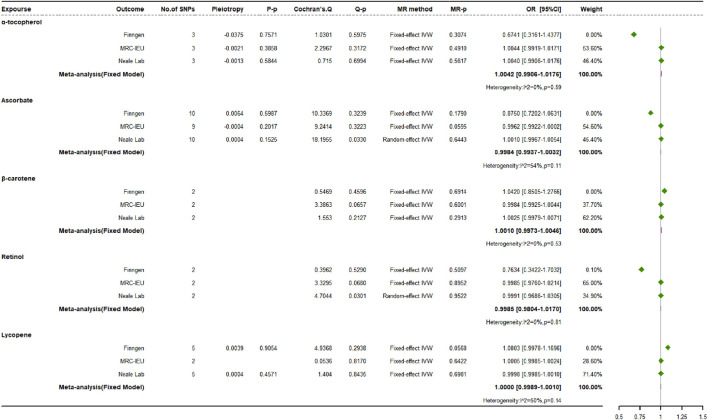
Causal impacts of exogenous antioxidants on osteoporosis risk.

The primary IVW analysis did not reveal any significant association between genetically derived absolute levels of exogenous antioxidants and the risk of OP in any of the three databases examined (Figure 5). For the MR analysis with only two SNPs, the results of the likelihood ratio method were generally consistent with the results of the main analysis. After natural log transformation, the OR per unit increment was 1.0010 (95% CI: 0.9973 to 1.0046) for *β*-carotene, 0.9985 (95% CI: 0.9804 to 1.0170) for retinol, 1.0000 (95% CI: 0.9989 to 1.0010) for lycopene per 1 *μ*g/dL, and 0.9984 (95% CI: 0.9937 to 1.0032) for ascorbate per 1 *μ*mol/L. For each 1 mg/dL increase in *α*-tocopherol, the OR was 1.0042 (95% CI: 0.9906 to 1.0176).

Multiple MR methods were used to assess the impact of three antioxidants with at least three SNPs, including ascorbate, *α*-tocopherol, and lycopene. The methods used included MR-Egger regression, weighted median estimation, simple mode, and weighted mode analyses. The results obtained from these methods were concordant. Although no significant heterogeneity was observed across studies using Cochran’s Q test for the association between exogenous antioxidants and OP, mild heterogeneity was detected during MR analyses when ascorbate and retinol data from the Neale Lab source were utilized. However, the instrumental SNPs showed no signs of directional pleiotropy, as evidenced by MR-Egger regression analyses. The MR-PRESSO tests confirmed that there is no horizontal pleiotropy affecting the relationship between exogenous antioxidant levels and OP risk. The conclusions were further strengthened by sensitivity analyses using a Leave-one-out strategy, which showed that the observed causal relationships were not dependent on any individual SNP. Therefore, these findings are robust to the influence of specific genetic alterations.

### 3.3 Causal impacts of exogenous antioxidant metabolites on osteoporosis risk

**FIGURE 6:**
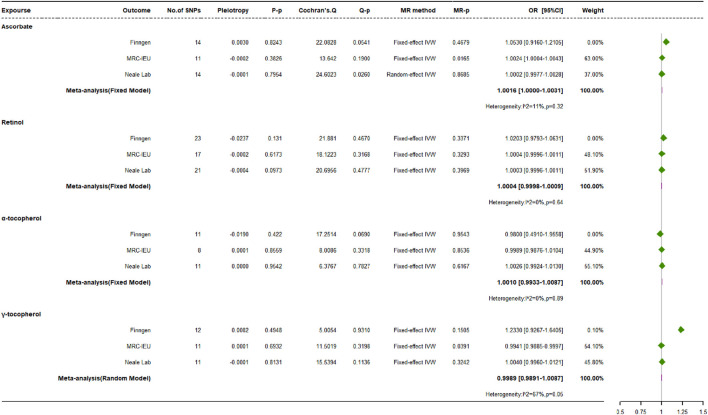
Causal impacts of exogenous antioxidant metabolites on osteoporosis risk.

Analogous conclusions emerged from our analysis of exogenous antioxidant metabolites (Figure 6), reinforcing the findings for their parent compounds. In-depth scrutiny across three distinct databases uniformly revealed that the majority of genetically regulated exogenous antioxidant metabolites displayed no substantial correlation with the occurrence of OP. A notable exception was discovered within the MRC-IEU dataset, suggesting that genetically elevated levels of *γ*-tocopherol might confer protection against OP (OR: 0.9941, 95% CI: 0.9885 to 0.9970, *p* = 0.0391, power = 3.8%).

Various MR techniques, such as MR-Egger, weighted median, simple mode, and weighted mode approaches, were used to confirm the associations between exogenous antioxidants and OP risk. Although Cochran’s Q statistic indicated heterogeneity in the relationship between ascorbate and OP when analyzing Neale Lab data, the remaining collective analyses showed consistency. No evidence of directional pleiotropy among the IVs was detected via MR-Egger regression, nor were anomalous SNPs indicative of horizontal pleiotropy uncovered by MR-PRESSO. Additionally, the leave-one-out sensitivity analysis, which excluded one SNP at a time, confirmed that the majority of the observed causal relations were not critically dependent on any singular genetic variant.

### 3.4 Causal impacts of endogenous antioxidants on osteoporosis risk

**FIGURE 7:**
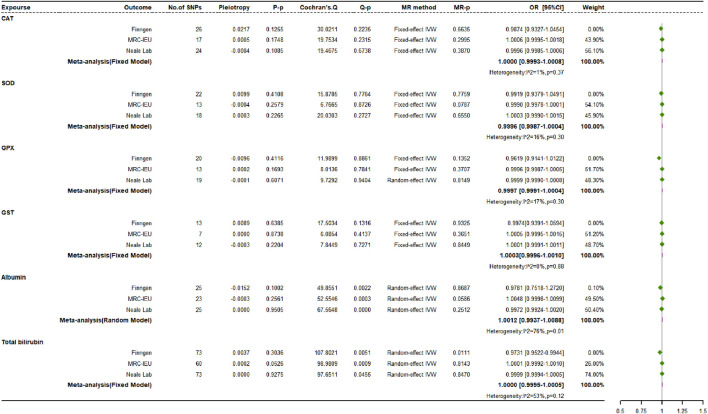
Causal impacts of endogenous antioxidants on osteoporosis risk.

Figure 7 shows the MR estimates for the causal relationships between six different endogenous antioxidants and OP. Analyses across three independent databases suggest that most genetically driven endogenous antioxidants do not have significant associations with OP risk. In the FinnGen study, an exception was observed where albumin showed a positive correlation with OP incidence (OR: 1.2587, 95% CI: 1.0009 to 1.5829, *p* = 0.0491, power = 8.3%), while total bilirubin demonstrated a potentially protective effect, inversely correlated with OP risk (OR: 0.9753, 95% CI: 0.9522 to 0.9990, *p* = 0.0411, power = 2.8%).

Using various MR techniques, including MR-Egger, weighted median, simple mode, and weighted mode strategies, we replicated the association between endogenous antioxidants and OP. Our results were consistent with the IVW approach. In the MR analyses of all three datasets, albumin, and serum total bilirubin exhibited heterogeneity, while GPX showed heterogeneity when analyzing OP data from the Neale Laboratory repository. The MR-Egger regression analyses did not reveal any evidence of directional multidirectionality between IVs. Using the FinnGen dataset, MR-PRESSO identified outlier SNPs for albumin (rs28929474) and total bilirubin (rs76895963) associated with OP susceptibility. After removing these outliers, updated MR analysis showed a change in the causal relationship between albumin and OP. This indicates that the previously observed positive correlation disappeared after removing potential bias (OR: 0.9781, 95% CI: 0.7518 to 1.272, *p* = 0.8687, power = 2.8%). Meanwhile, the OR estimate for total bilirubin remained statistically significant with no significant change (OR: 0.9731, 95% CI: 0.9522 to 0.9944, *p* = 0.0111, power = 2.9%). Furthermore, leave-one-out sensitivity analysis confirmed that most of the causal associations were not solely determined by individual genetic variations.

### 3.5 Causal impact of osteoporosis on endogenous antioxidants

**FIGURE 8:**
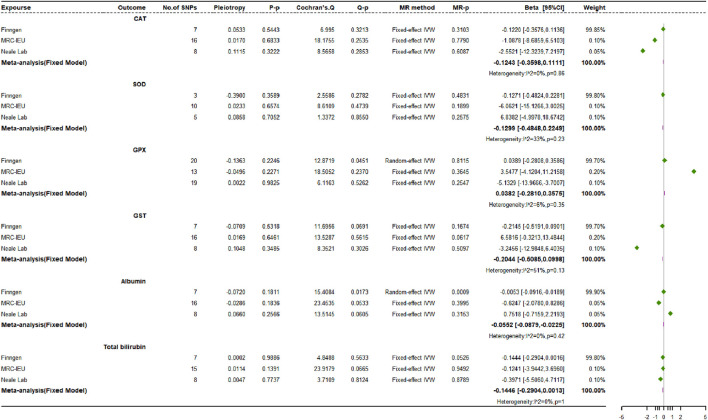
Causal impact of osteoporosis on endogenous antioxidants.

In the bidirectional MR analysis, we observed a statistically significant causal effect of OP on albumin (*β*: −0.0552, 95% CI: −0.0879 to −0.0225, *p* = 0.0009, power = 100%), which maintained significance even after adjusting for the Bonferroni correction threshold (Figure 8). However, we did not identify any causal connections between OP and the other endogenous antioxidants under scrutiny.

Various MR methods, such as MR-Egger regression, the weighted median technique, simple mode, and weighted mode evaluations, were used to investigate the potential causal link between OP and endogenous antioxidants. The results were consistent with those obtained from the IVW method. Statistical heterogeneity, as evaluated by Cochran’s Q test, was significant only in the relationship between OP cases sourced from the FinnGen dataset and the biomarkers GPX and albumin. Other genetic and phenotypic correlation analyses did not provide enough evidence to infer heterogeneity. It is important to note that MR-PRESSO analysis did not detect any outlier SNPs that could plausibly cause horizontal pleiotropy. It should be emphasized that the leave-one-out sensitivity assessment robustly confirmed that most observed causal relationships were not exclusively contingent upon any specific genetic polymorphism.

## 4 Discussion

In the present research, we utilized a dual-sample MR strategy to conduct a comprehensive examination of the causal relationship between both extrinsic and intrinsic antioxidants and OP. Despite the results indicating that neither the consumption of exogenous antioxidants, which include ascorbate, tocopherol, carotenoids, retinol, and lycopene, nor augmented activities within the intrinsic antioxidant defense system, composed of enzymes such as CAT, SOD, GPX, GST, serum albumin, and total bilirubin, showed a statistically discernible causal link with OP risk, our study disclosed a noteworthy observation suggesting that OP itself might act as a causative agent contributing to reduced total albumin concentrations. Additionally, exploratory analyses of data from the FinnGen and MRC-IEU repositories suggested a potential beneficial role of genetically high *γ*-tocopherol levels and increased serum total bilirubin in mitigating OP risk. Nonetheless, when extending our analysis through a meta-analysis incorporating data from three separate databases, we did not obtain uniform supportive evidence across these larger datasets.

In the course of biological activities, especially within cellular energy metabolism and the biosynthesis of adenosine triphosphate (ATP), the production of ROS as a byproduct of oxygen reduction is unavoidable. An overabundance of ROS can provoke oxidative stress, a condition that is broadly accepted as a key contributor to the advancement of a broad spectrum of age-related and degenerative diseases, OP being one among them (León-Reyes et al., 2023). A multitude of studies highlights the core position occupied by oxidative stress in the senescence process and the pathophysiology of OP. Antioxidants serve a vital protective function by countering ROS, preserving cellular redox equilibrium, and thereby effectively shielding cells from oxidative injury caused by ROS, thereby offering the potential for suppressing the development of OP (Damani et al., 2022; Davan et al., 2023).

Albumin is the most abundant protein in plasma and serves as an important endogenous antioxidant reservoir in response to chronic oxidative stress (Atukeren et al., 2010). Its antioxidant properties are mainly derived from the exposed thiol moiety at the Cys34 site. During oxidative stress, Cys34 can form disulfide bridges with free cysteine or glutathione, which alters the tertiary structure of human serum albumin (HSA) and effectively eliminates ROS such as hydrogen peroxide, peroxynitrite, superoxide radicals, and hypochlorous acid (Kawakami et al., 2006; Belinskaia et al., 2021). Previous observational research has presented varying perspectives on the relationship between albumin and OP risk. For instance, in a large study of 21,121 individuals with a mean age of 61 years, Afshinnia et al. found that higher serum albumin levels were negatively correlated with OP prevalence, indicating a decline in OP incidence with rising albumin (Afshinnia et al., 2016). In contrast, Rico et al. discovered in a case-control study that serum albumin levels were significantly lower in elderly women with OP compared to healthy counterparts (Rico et al., 2009). However, some studies have questioned the independent correlation between serum albumin and BMD. For instance, a study conducted in Rancho Bernardo reported a slight positive correlation between serum albumin and BMD, which disappeared after adjusting for age (Lunde et al., 1998). On the other hand, a different study conducted on healthy postmenopausal women did not find any significant correlation between serum albumin and BMD (D’Erasmo et al., 1999). The preliminary analysis of the FinnGen database in the current study suggests a potential positive association between albumin and the onset of OP. However, after removing outliers using MR-PRESSO, the causal relationship between albumin and OP no longer had statistical significance. The reverse MR analysis revealed a new causal pathway, indicating that OP may reduce serum albumin levels. It is hypothesized that this reduction in albumin levels may be linked to oxidative stress, poor nutritional status, and chronic inflammation during the course of OP (Vaughan et al., 2012; Cauley et al., 2016; Tugba et al., 2019; Xu et al., 2022). Extensive research has highlighted the significant role of oxidative stress in the pathogenesis of osteoporosis (Manolagas, 2010; Zhang et al., 2023). Oxidative stress has been identified as a significant contributor to hypoalbuminemia (Chojkier, 2005), with the potential to induce molecular modifications in human serum albumin, including carbonylation and the formation of advanced oxidation protein products and advanced glycation end-products (Michelis et al., 2010). Studies have demonstrated that the cobalt binding capacity of albumin is diminished in individuals diagnosed with osteoporosis, reflecting oxidative damage and a decline in albumin functionality (Tugba et al., 2019). Albumin, an essential protein in the bloodstream, is significantly correlated with muscle mass and strength in elderly individuals with osteoporosis, suggesting a potential nutritional link (Xu et al., 2022). Poor nutritional status is prevalent among the elderly, particularly those diagnosed with osteoporosis, which may contribute to hypoalbuminemia (Xu et al., 2022). Additionally, the synthesis of albumin may be inhibited due to the body’s response to inflammation (Vaughan et al., 2012; Cauley et al., 2016). Overall, our study provides new insights for clinical practice by establishing a causal link between serum albumin and OP. Monitoring changes in serum albumin levels could serve as a biomarker for evaluating disease progression and treatment responsiveness in OP (Fujii et al., 2018). In the future clinical handling of OP, measuring serum albumin levels could provide valuable information for early disease risk detection, tracking disease progression, and devising personalized therapeutic regimens, in addition to standard bone density measurements. However, this proposition requires further substantiation through extensive prospective studies and clinical trials to verify its practicality and effectiveness in OP management.

Bilirubin was previously considered a cytotoxic byproduct. Despite this, bilirubin has been shown to hinder osteoblast function, including growth, differentiation, and mineralization (Turkeli et al., 2008). Recent research, however, has shown that it has antioxidant potential, sometimes even surpassing certain vitamin E analogs in combating lipid peroxidation (Wu et al., 1994; Žiberna et al., 2021). A cross-sectional study of 918 postmenopausal women found a positive correlation between elevated total bilirubin levels and improved BMD as well as reduced risk of osteoporosis (Bian et al., 2013). The impact of *α*-tocopherol and *γ*-tocopherol, which are key forms of vitamin E found in the human body and diet and possess antioxidant and anti-inflammatory qualities, on skeletal metabolism remains unclear (Macdonald et al., 2004; Hamidi et al., 2012). MacDonald et al.’s research suggests that excess vitamin E intake may negatively correlate with BMD (Macdonald et al., 2004). However, another study suggests that *γ*-tocopherol may promote bone formation in postmenopausal women by modulating bone turnover processes (Hamidi et al., 2012). Our study, which utilized MR analyses on FinnGen and MRC-IEU datasets, has preliminarily discovered a potentially significant association between genetically higher levels of *γ*-tocopherol and serum total bilirubin levels, and a reduced risk of OP. This suggests a positive role for these compounds in bone health, potentially aiding in the prevention of OP due to their potent antioxidant and anti-inflammatory characteristics. Although our FinnGen and MRC-IEU analyses revealed an association, attempts to generalize this finding across multiple independent databases through a meta-analysis did not yield strong and consistent statistical support. This inconclusiveness could result from several factors, including population-specific genetic heterogeneity, sample size constraints, potential confounders, and complex interplays between environmental factors and study outcomes.

In recent MR investigations, researchers have not been able to establish a significant causal link between circulating bilirubin concentrations and either extraskeletal bone mineral density (eBMD) or the incidence of fractures. This suggests that bilirubin may not be a direct, pivotal factor affecting OP susceptibility (Zhao et al., 2021). Li’s study suggests that antioxidants *β*-carotene and *γ*-tocopherol may potentially mitigate OP risk. However, the robustness of these claims requires more rigorous examination. Li’s study used a meta-analytic approach to enhance the persuasiveness of the conclusions. However, the credibility and broad applicability of these outcomes were limited by reliance on just two discrete datasets. Of particular relevance, the extensive osteoporosis-centric research endeavor, the GEFOS database, did not reveal any statistically discernible disparities in OP risk between *β*-carotene and *γ*-tocopherol levels (Li et al., 2023).

In this research, we leveraged the extensive capabilities of large-scale MR techniques, utilizing aggregated data from GWAS involving 1,178,298 cases and controls, to pioneer a systematic inquiry into the potential causal links between exogenous antioxidants—those obtained through dietary supplements or pharmaceutical ingestion—and endogenous antioxidants with the risk of OP. A prime virtue of our methodology is the employment of genetic proxies instead of conventional interventions, thereby mitigating potential risks associated with direct antioxidant administration and mitigating issues of reverse causality between exposures and outcomes, thus enhancing the precision of causal reasoning. Additionally, the GWAS subjects in this investigation were predominantly of European descent, and we applied meticulous genomic control procedures uniformly across all studies. This greatly diminishes biases resulting from population stratification and genomic inflation effects, thereby strengthening the internal validity of our findings. In pursuit of further validation and consistency, we conducted a meta-analysis of data from three major databases. Our statistical heterogeneity assessments demonstrated that the merged findings across these three databases displayed a relatively low degree of heterogeneity, which partly attests to the robustness of our study’s conclusions.

In this study, we employ MR to explore the causal relationship between antioxidants and the risk of OP. However, it is crucial to acknowledge several limitations. Firstly, one significant limitation not addressed directly in our analysis is the intake of exogenous antioxidants, which was not measured through dietary consumption frequencies or 24-h dietary recalls. This omission might have influenced the observed associations, as dietary antioxidant intake could modulate the effects of genetically predicted antioxidant levels. Future studies should incorporate detailed dietary assessments to better control for this potential confounder. Secondly, the selection of single nucleotide polymorphisms (SNPs) in our study involved different criteria, including various *p*-value thresholds (e.g., *p* $<\;5\times 10^{-8}$ , *p* $<\;5\times 10^{-6}$ , *p* $<\;1\times 10^{-5}$ ) and different LD thresholds (e.g., LD < 0.02 and LD < 0.001). The variation in the selection of SNPs was due to the limited availability of reliable genetic instruments for the antioxidants under investigation. As larger sample GWAS for exposure and outcomes emerge, this limitation may diminish. Thirdly, the current study exclusively utilized GWAS data for OP and did not include BMD measurements from the forearm, hip, or whole body. This approach may restrict the breadth of our osteoporosis risk assessment. Given that BMD is a pivotal diagnostic marker for osteoporosis, and measurements from different anatomical sites can offer varied insights into bone health, the omission of comprehensive BMD data could impact the accuracy of our findings. Future research should incorporate BMD measurements from multiple sites to enhance the thoroughness of risk assessment and more precisely determine which anatomical site’s BMD most effectively predicts OP risk. Fourthly, the relationship between osteoporosis and reduced serum albumin concentrations remains complex and not fully elucidated in the current literature. While some studies suggest a potential link between the two, others have not found direct evidence supporting osteoporosis as a causative factor in lowering serum albumin levels. Consequently, further investigations are warranted to clarify the causal relationship and clinical significance between osteoporosis and reduced serum albumin levels. Finally, the generalizability of our findings to other racial and ethnic groups may be constrained, as the GWAS data primarily comprised individuals of European descent.

## 5 Conclusion

This MR study used a large dataset and rigorous genetic analysis to provide substantial evidence for exploring the causal relationship between antioxidants and OP risk in Europeans. Although the study could not definitively confirm a statistically significant causal link between exogenous and endogenous antioxidants and OP risk, it reveals that osteoporosis may contribute to reduced serum albumin levels. This suggests that a decrease in serum albumin levels may be strongly associated with the progression of OP. Furthermore, this study indicates that monitoring dynamic fluctuations in serum albumin levels can serve as an effective biomarker for evaluating treatment efficacy and tracking the progression of OP in clinical practice.

## Data Availability

The original contributions presented in the study are included in the article/Supplementary Material, further inquiries can be directed to the corresponding authors.
