# Supplementary material for: Exogenous and endogenous antioxidants in osteoporosis risk: causal associations unveiled by Mendelian Randomization analysis
Source: Front Physiol. 2024 May 31;15:1411148. doi: 10.3389/fphys.2024.1411148 (PMC11176562; doi:10.3389/fphys.2024.1411148)
Supplement: Supplementary file 1 [file DataSheet1.pdf]

frontiers

Supplementary Material

1 SUPPLEMENTARY TABLES AND FIGURES

1.1 Figures

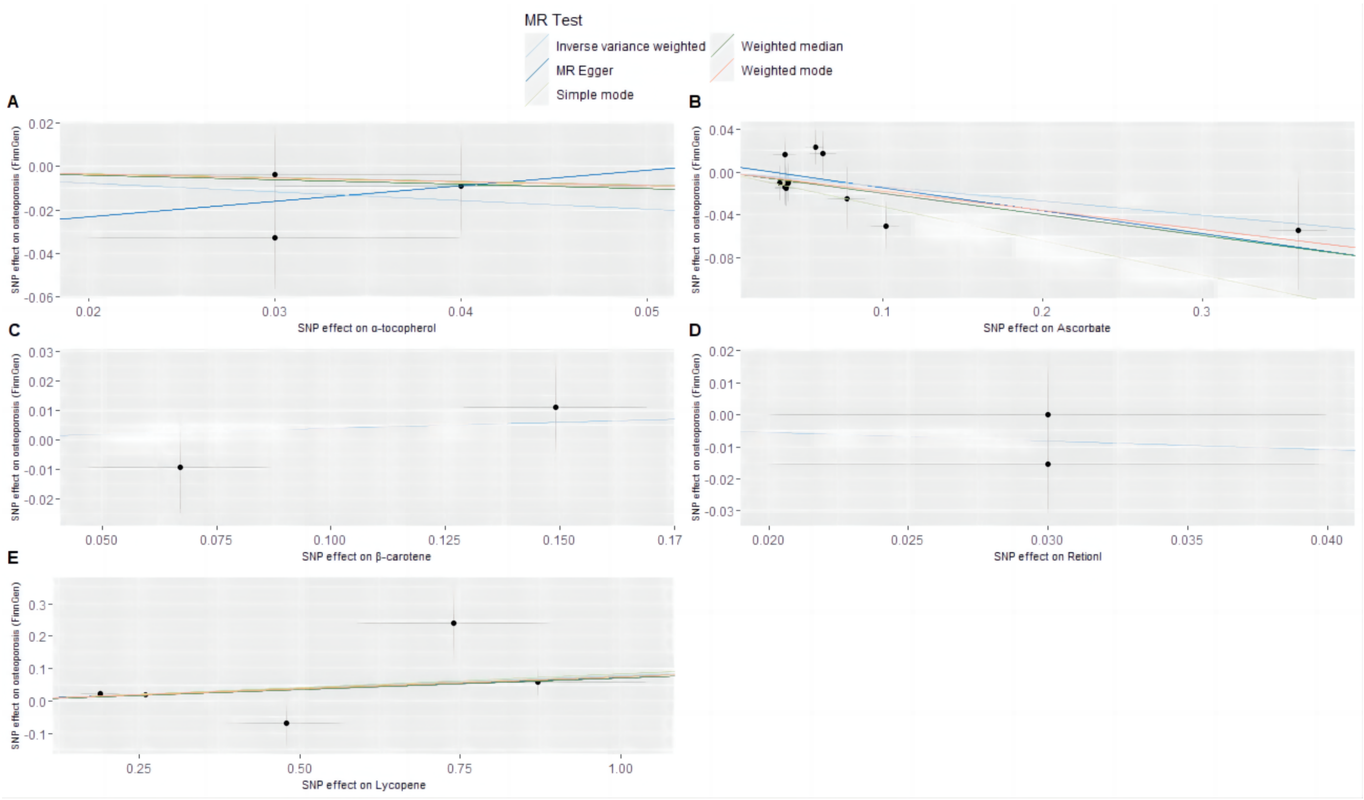

Figure S1. Scatter plot:Exogenous Antioxidants on Osteoporosis(FinnGen)

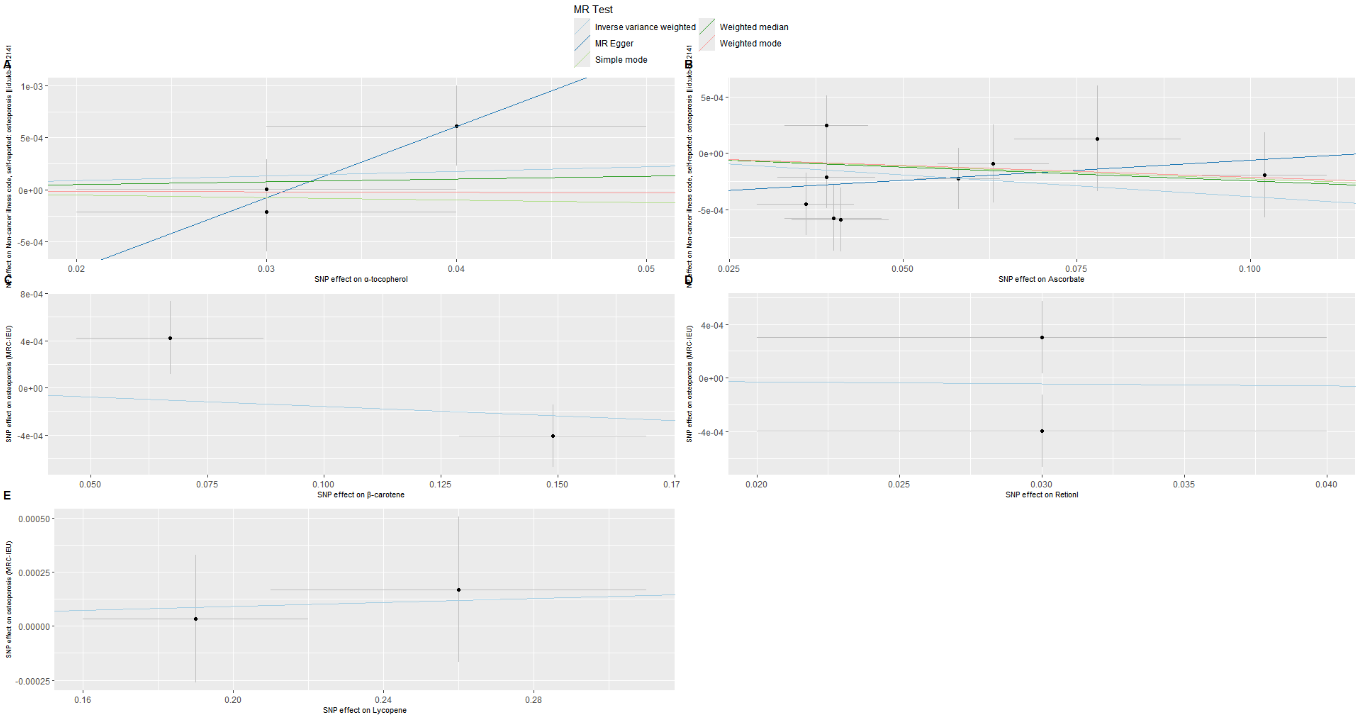

Figure S2. Scatter plot:Exogenous Antioxidants on Osteoporosis(MRC-IEU)

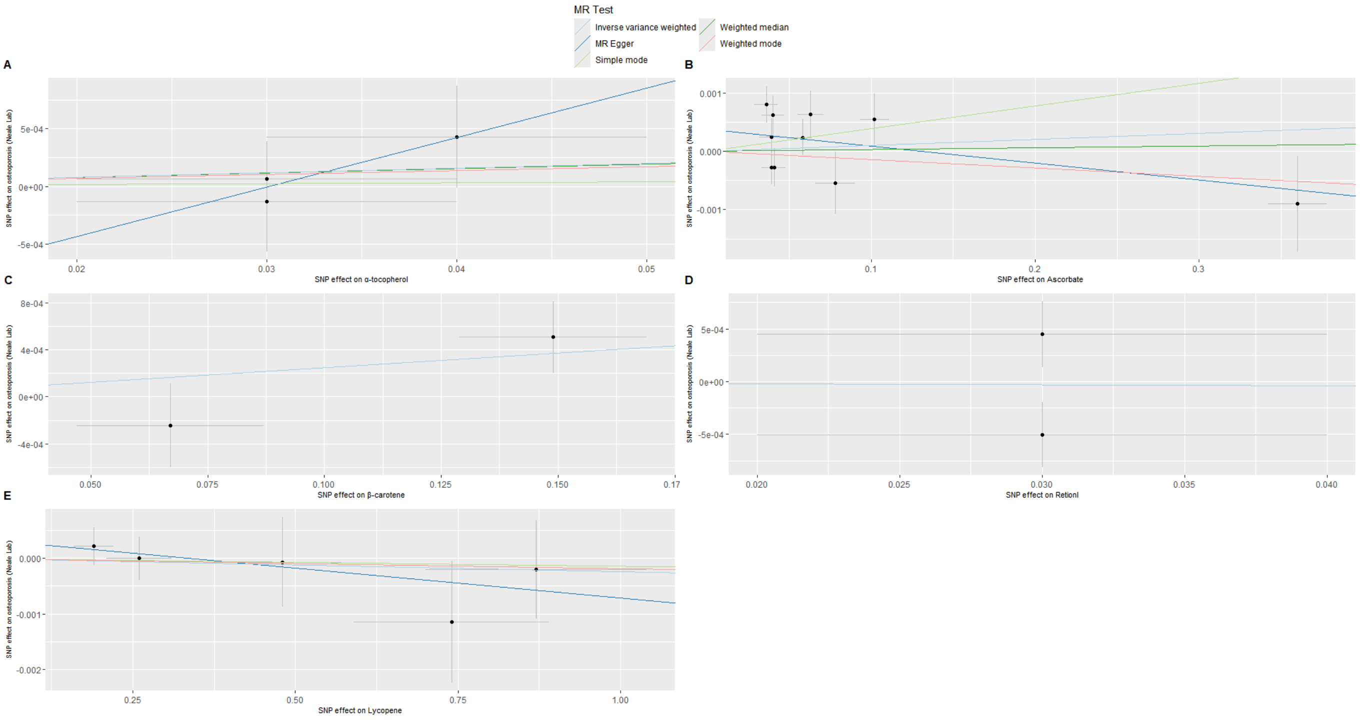

Figure S3. Scatter plot:Exogenous Antioxidants on Osteoporosis(Neale Lab)

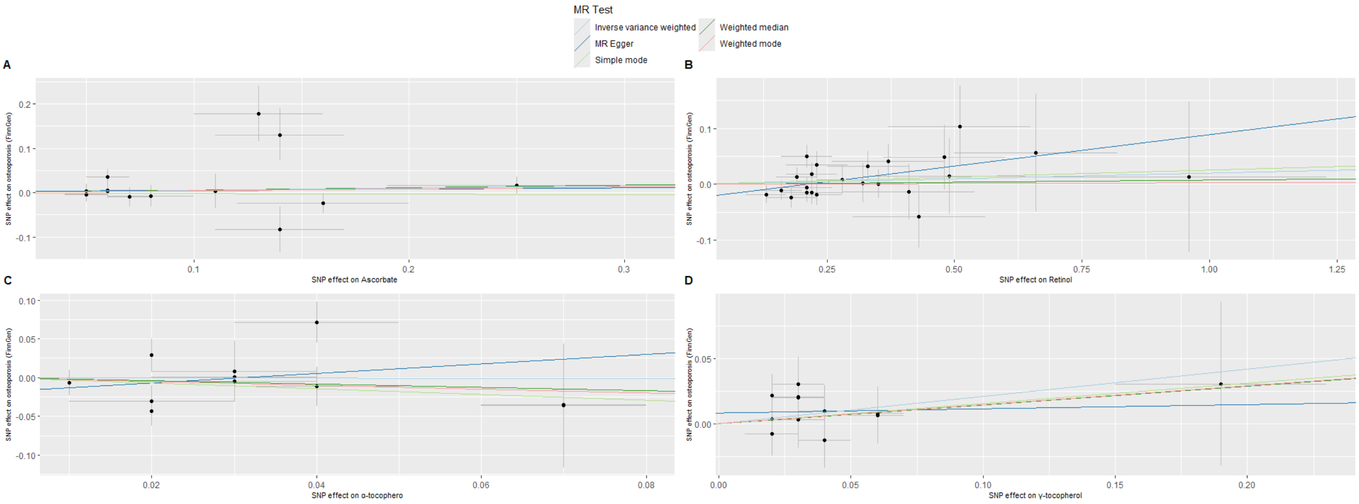

Figure S4. Scatter plot:Exogenous Antioxidant Metabolites on Osteoporosis(FinnGen)

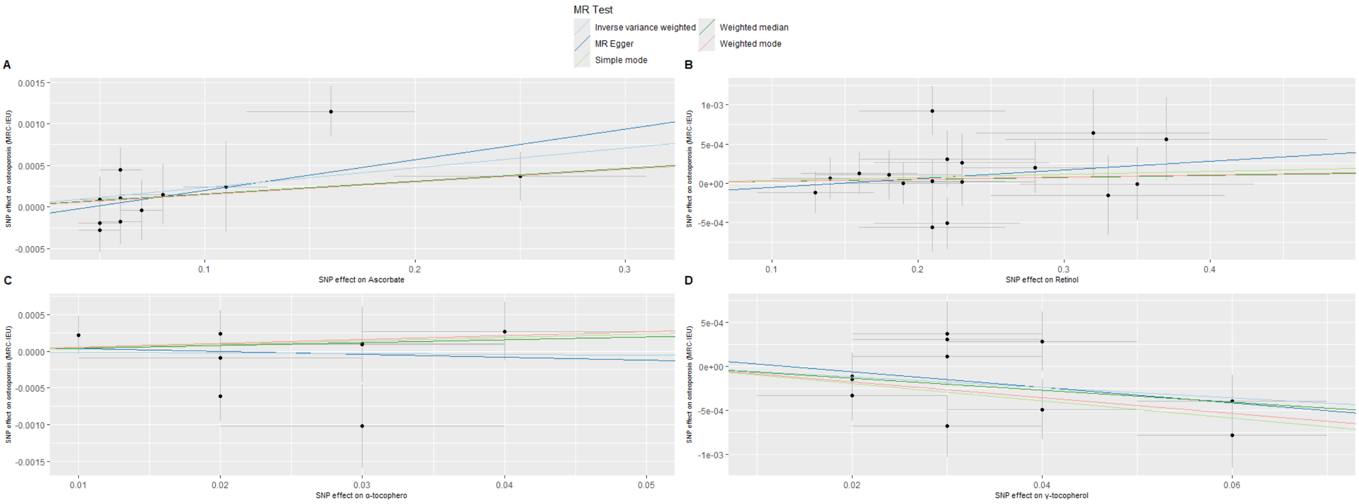

Figure S5. Scatter plot:Exogenous Antioxidant Metabolites on Osteoporosis(MRC-IEU)

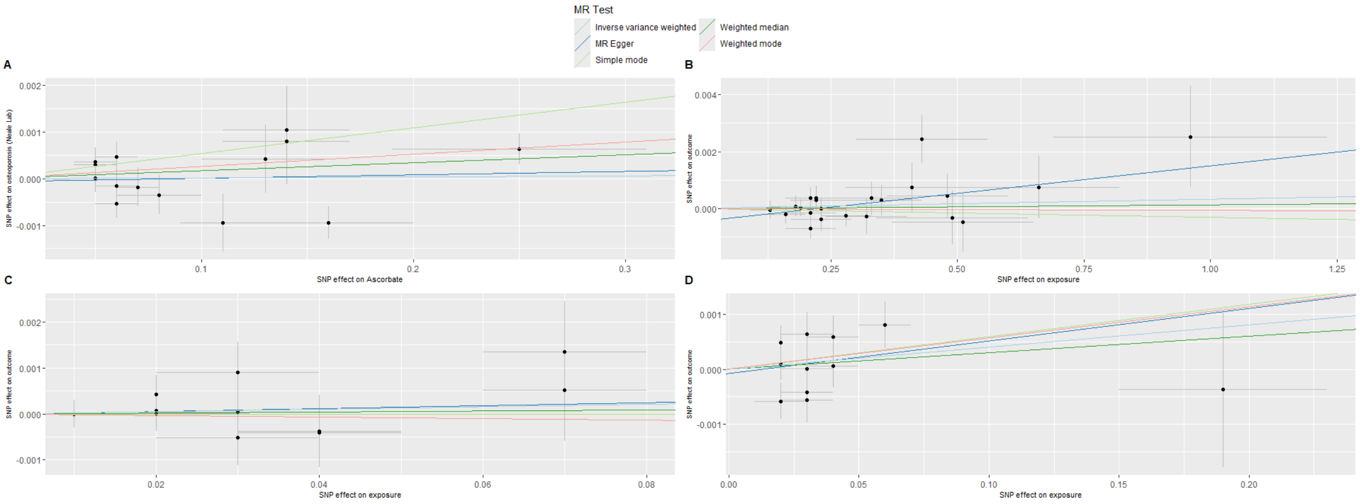

Figure S6. Scatter plot:Exogenous Antioxidant Metabolites on Osteoporosis(Neale Lab)

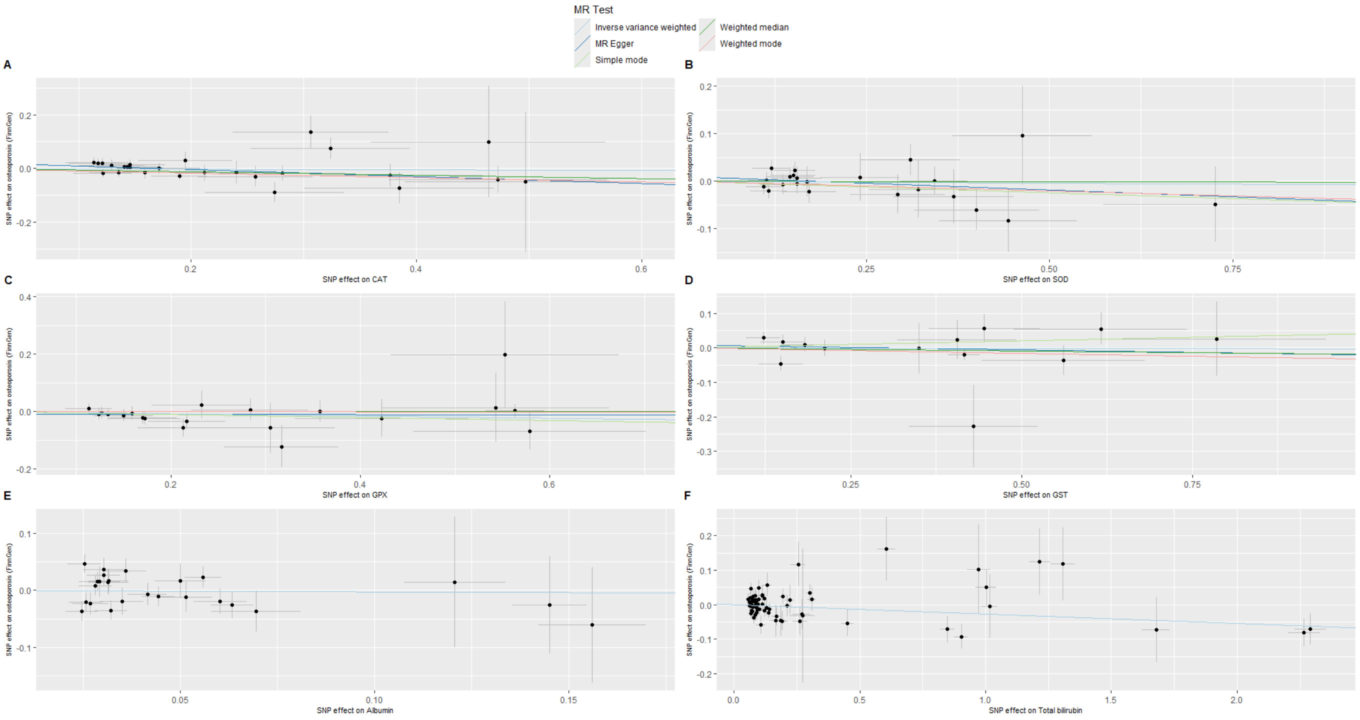

Figure S7. Scatter plot:Endogenous Antioxidants on Osteoporosis(FinnGen)

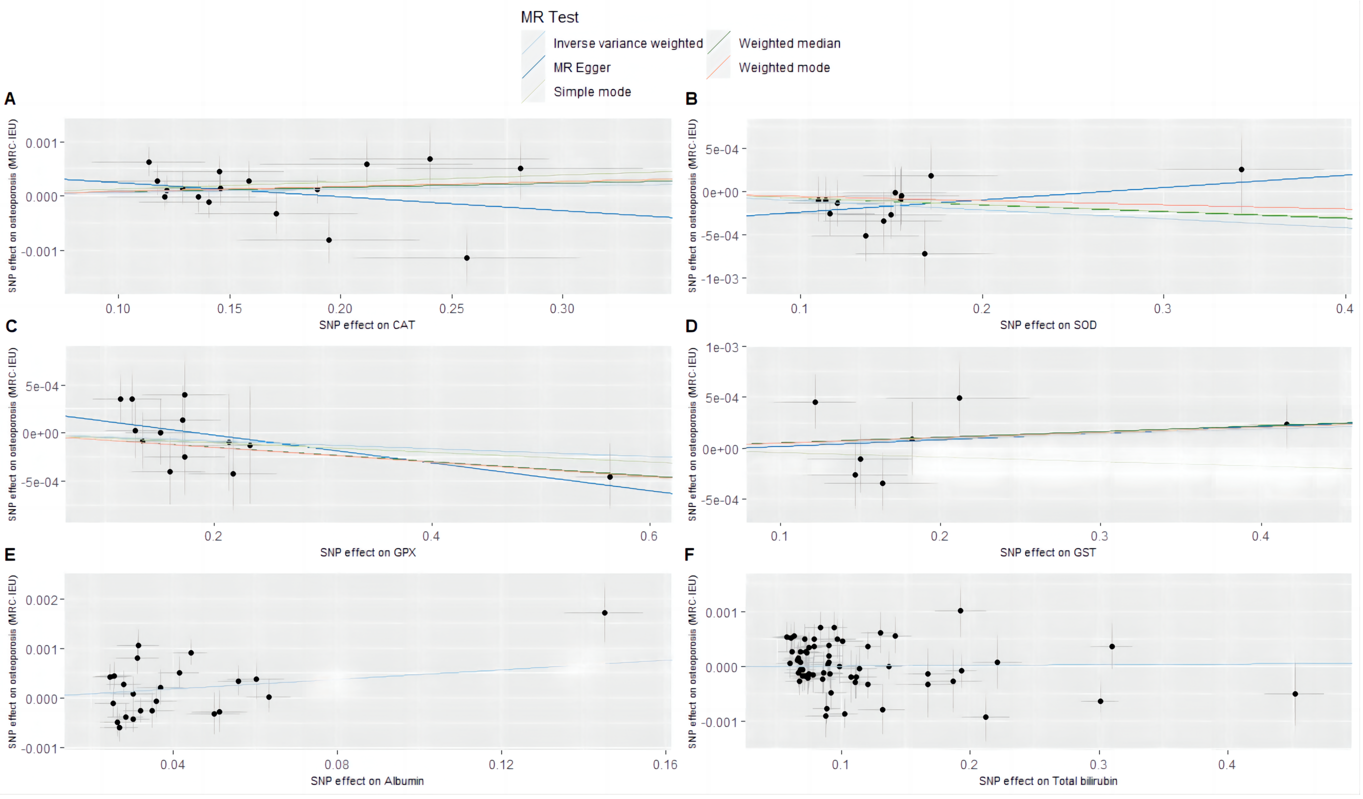

Figure S8. Scatter plot:Endogenous Antioxidants on Osteoporosis(MRC-IEU)

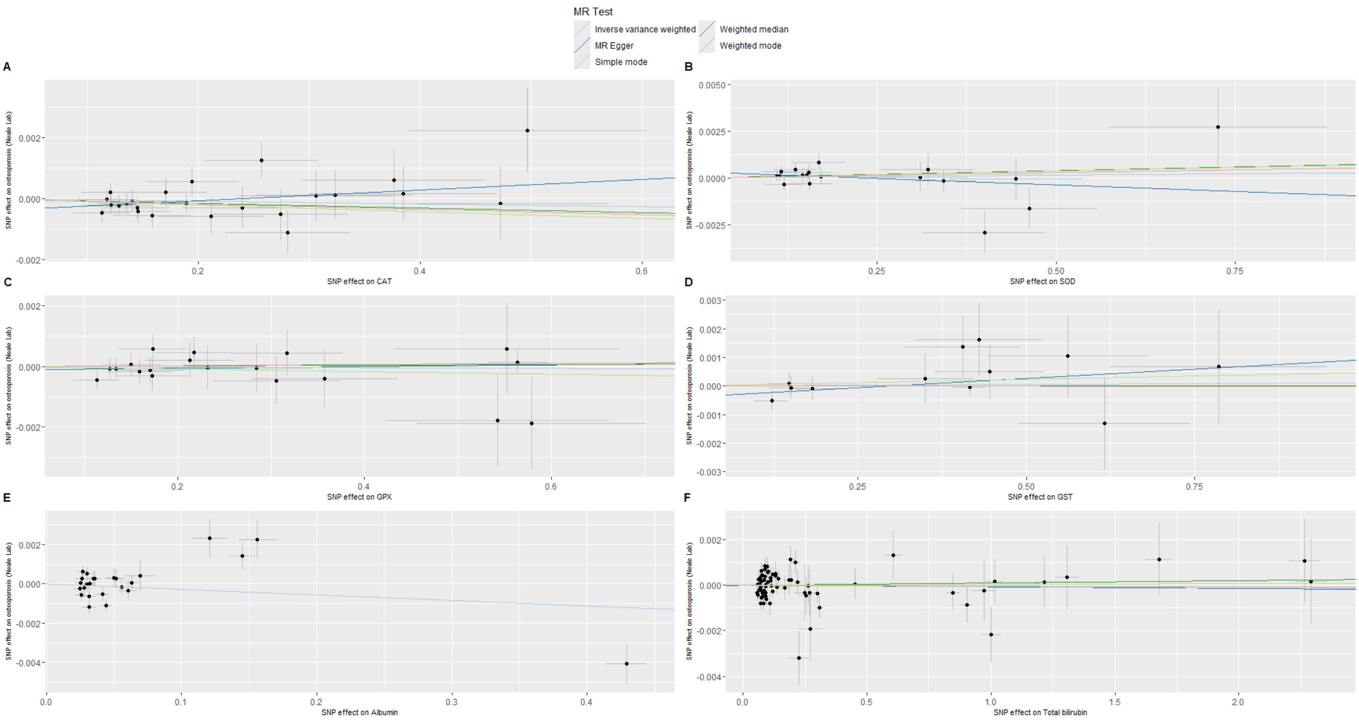

Figure S9. Scatter plot:Endogenous Antioxidants on Osteoporosis(Neale Lab)

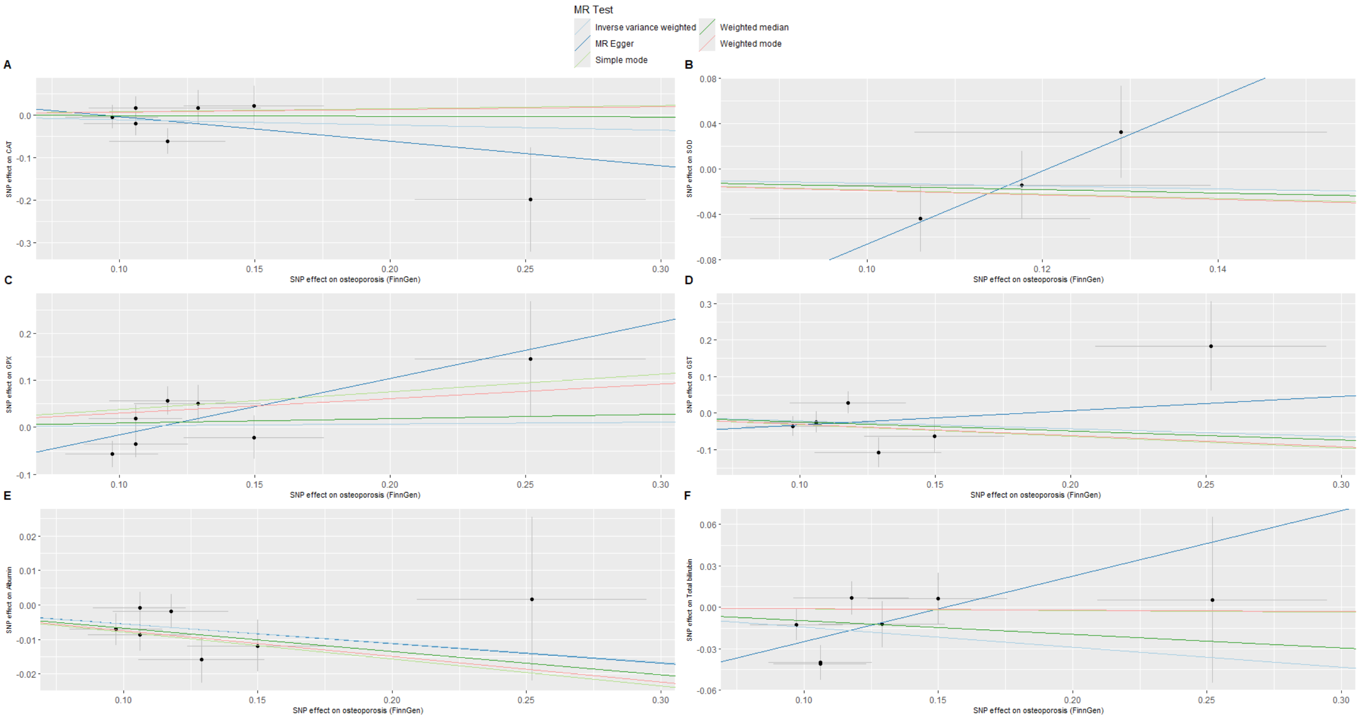

Figure S10. Scatter plot:Osteoporosis on Endogenous Antioxidants (FinnGen)

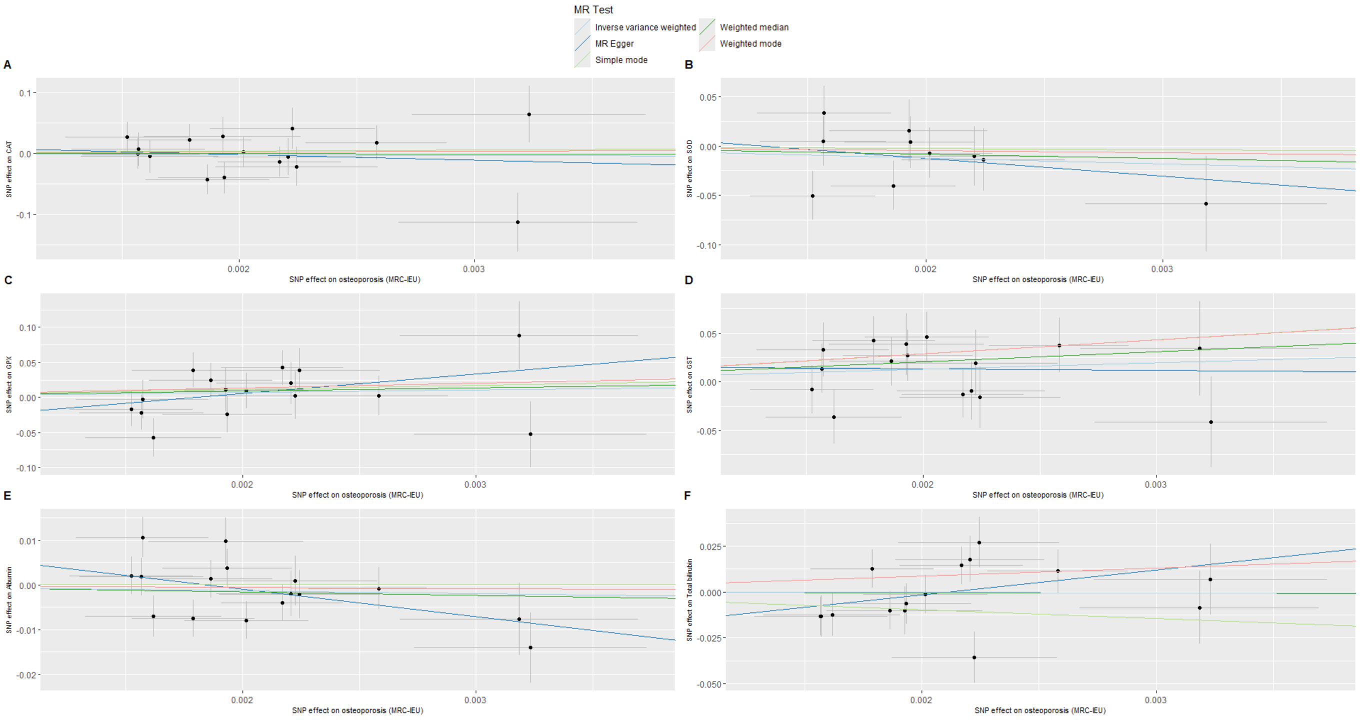

Figure S11. Scatter plot:Osteoporosis on Endogenous Antioxidants (MRC-IEU)

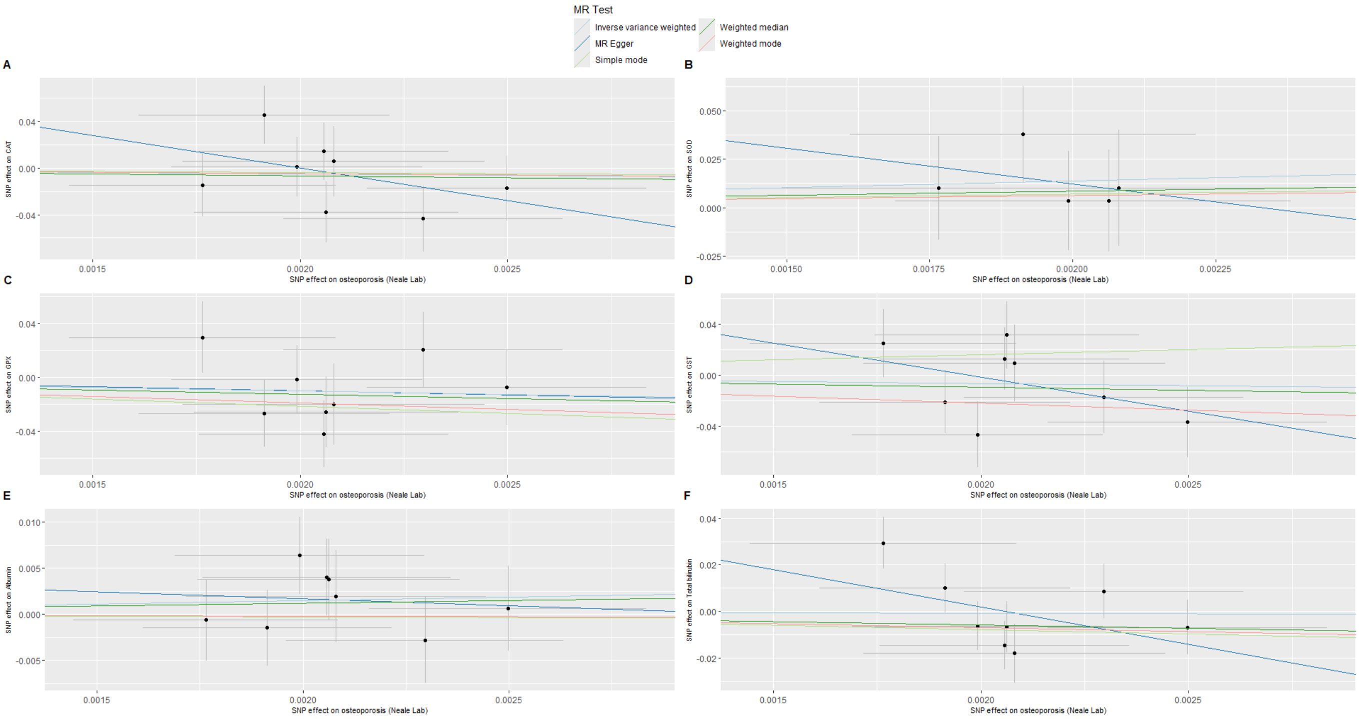

Figure S12. Scatter plot-Osteoporosis on Endogenous Antioxidants (Neale Lab)

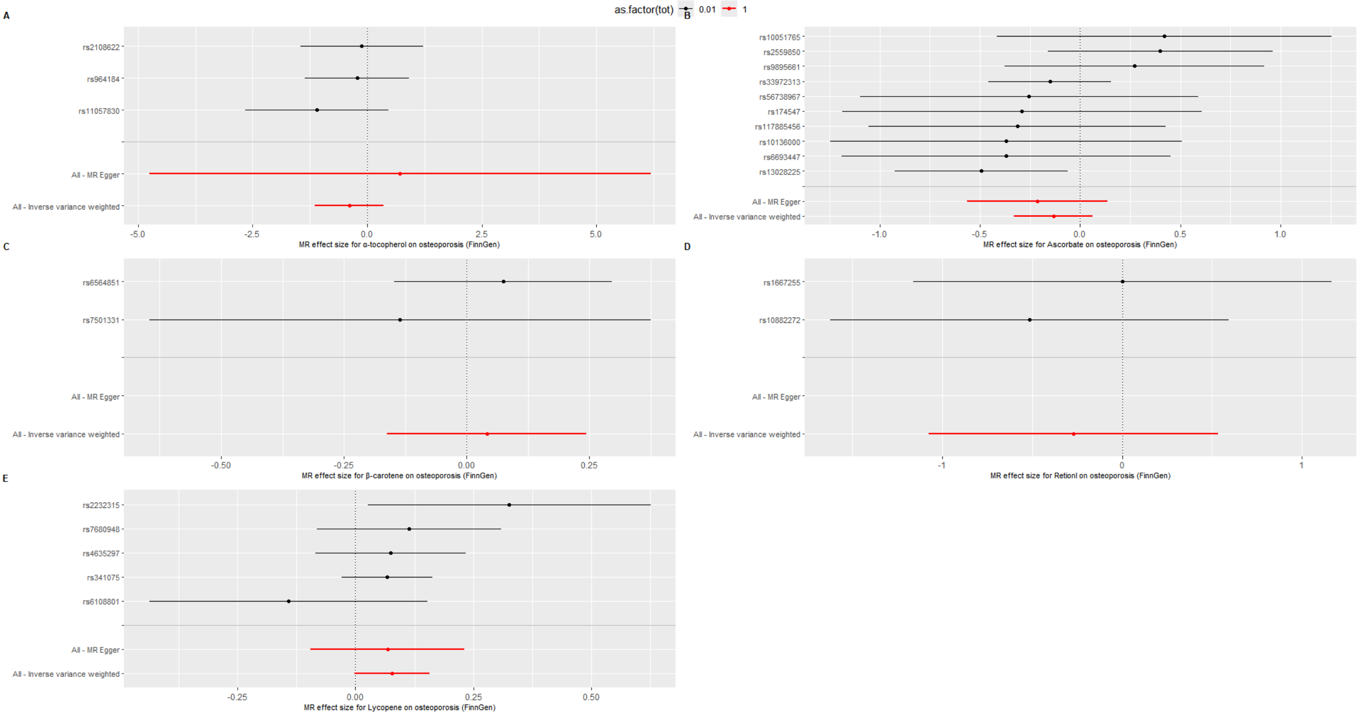

Figure S13. Forest plot:Exogenous Antioxidants on Osteoporosis(MRC-IEU)

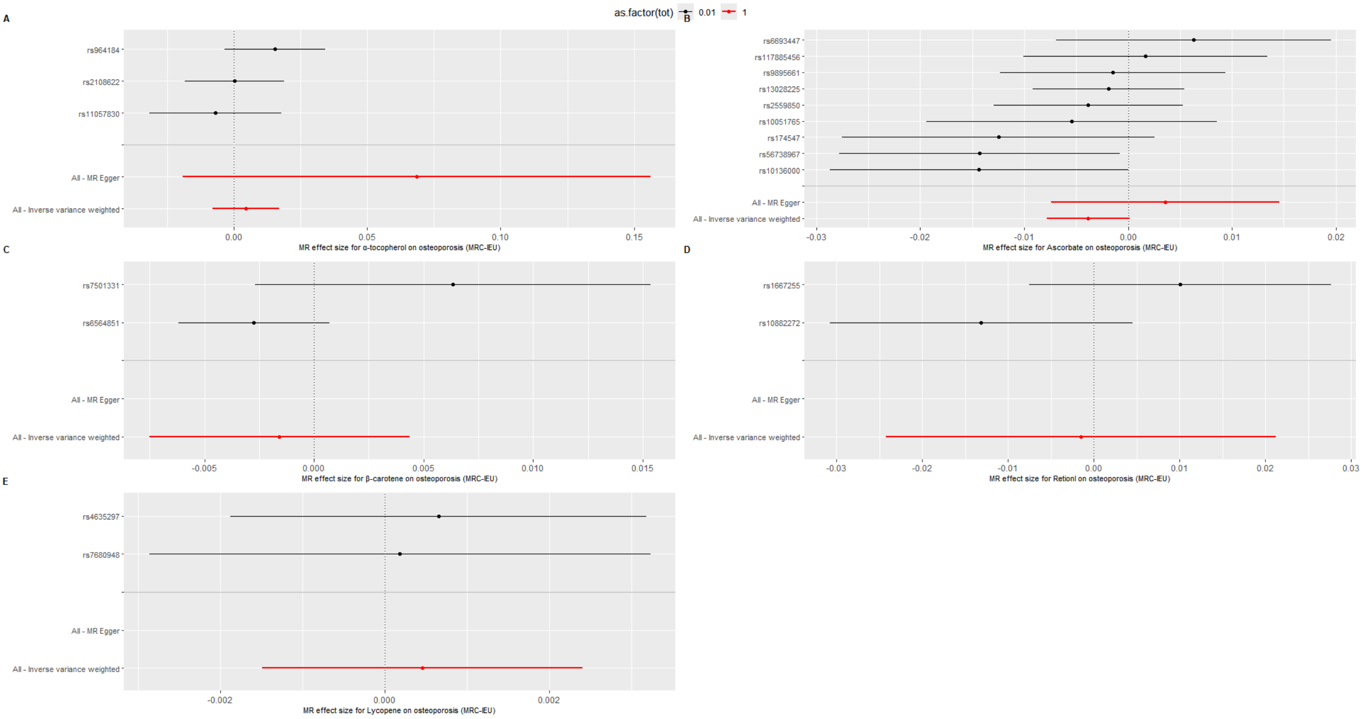

Figure S14. Forest plot:Exogenous Antioxidants on Osteoporosis(Neale Lab)

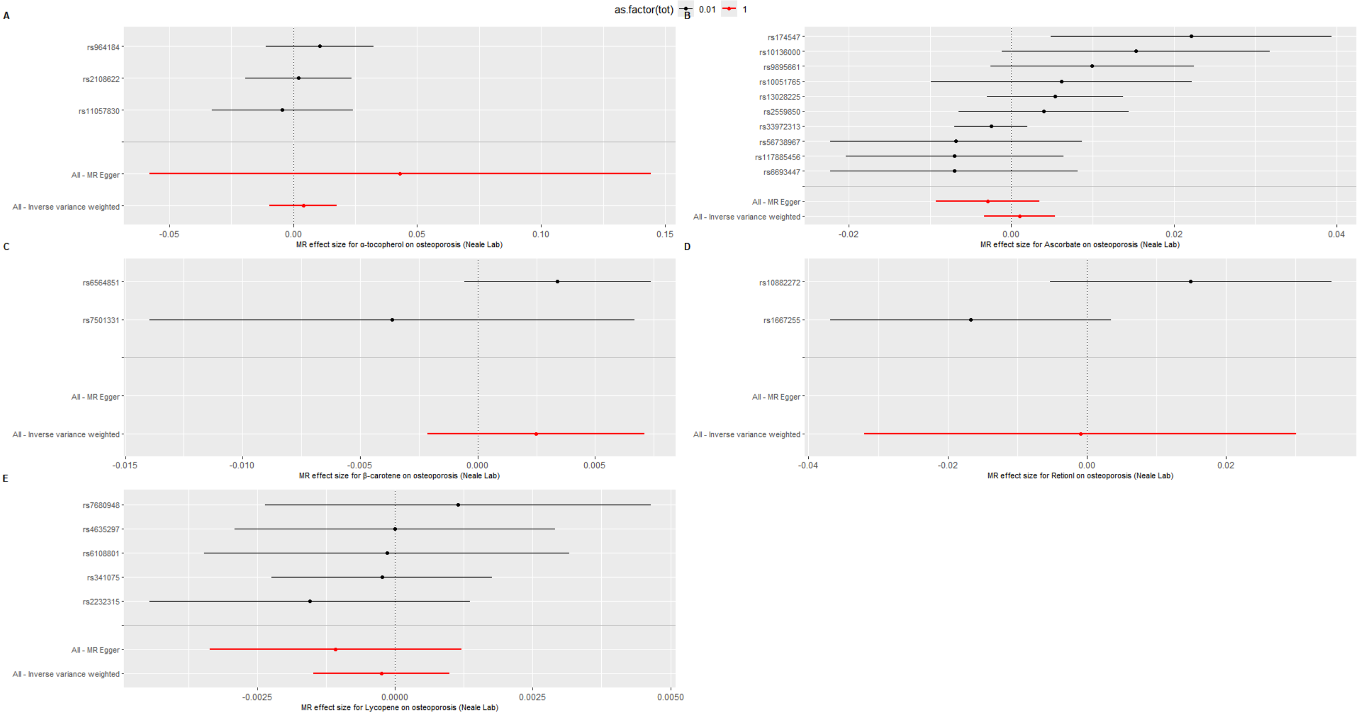

Figure S15. Forest plot:Exogenous Antioxidant Metabolites on Osteoporosis(FinnGen)

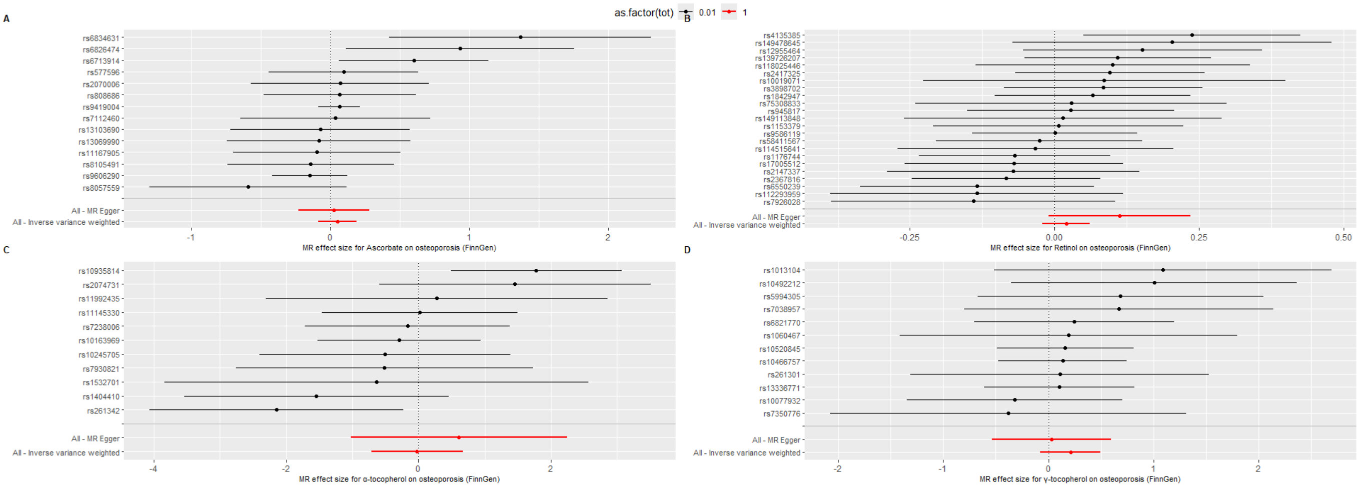

Figure S16. Forest plot:Exogenous Antioxidant Metabolites on Osteoporosis(MRC-IEU)

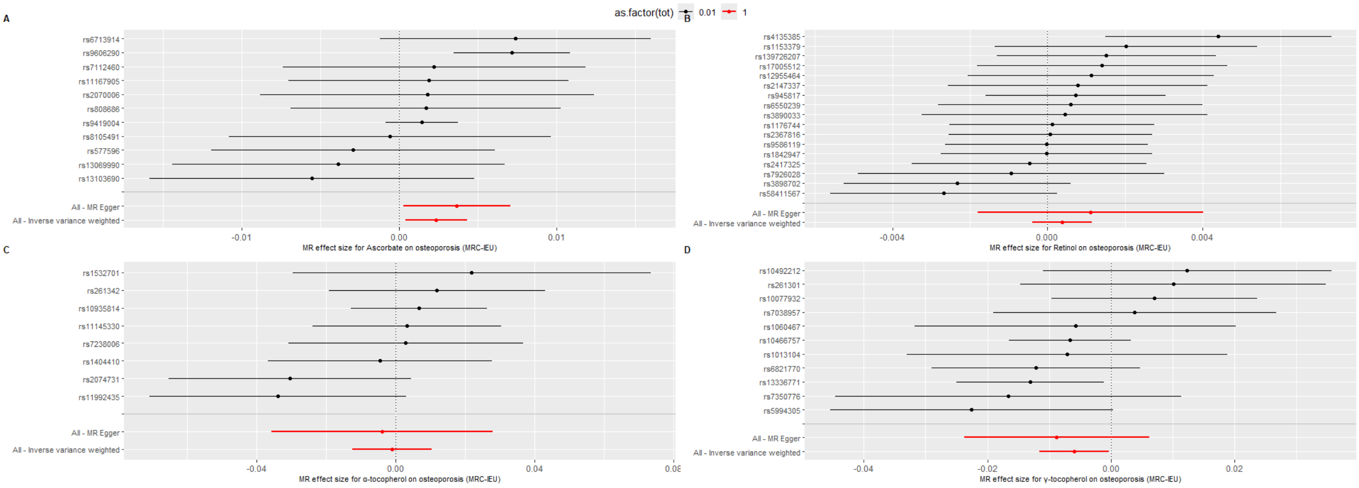

Figure S17. Forest plot:Exogenous Antioxidant Metabolites on Osteoporosis(Neale Lab)

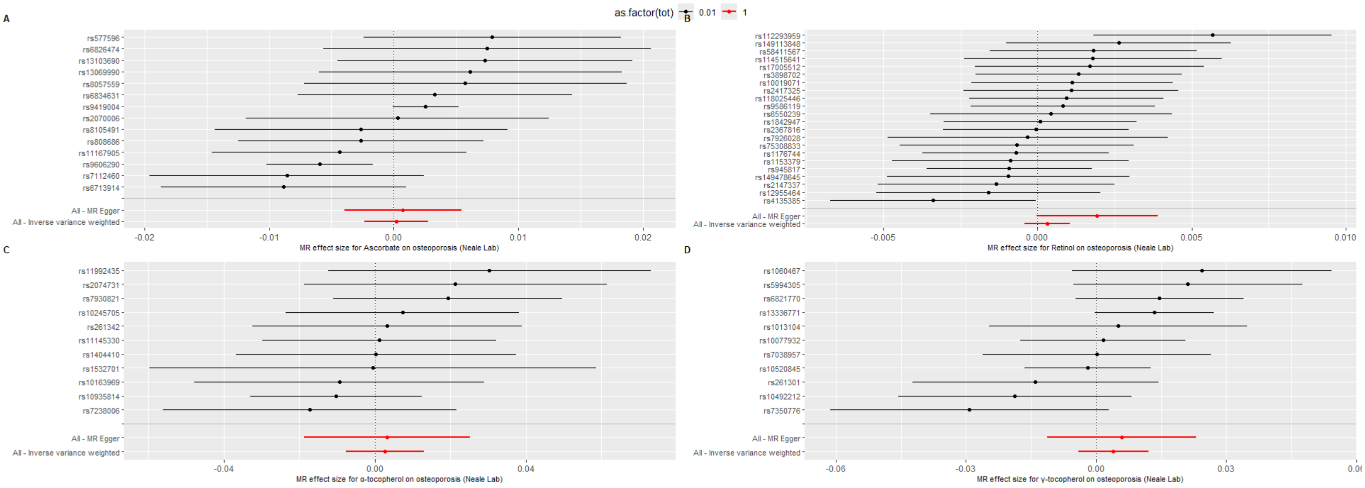

Figure S18. Forest plot:Endogenous Antioxidants on Osteoporosis(FinnGen)

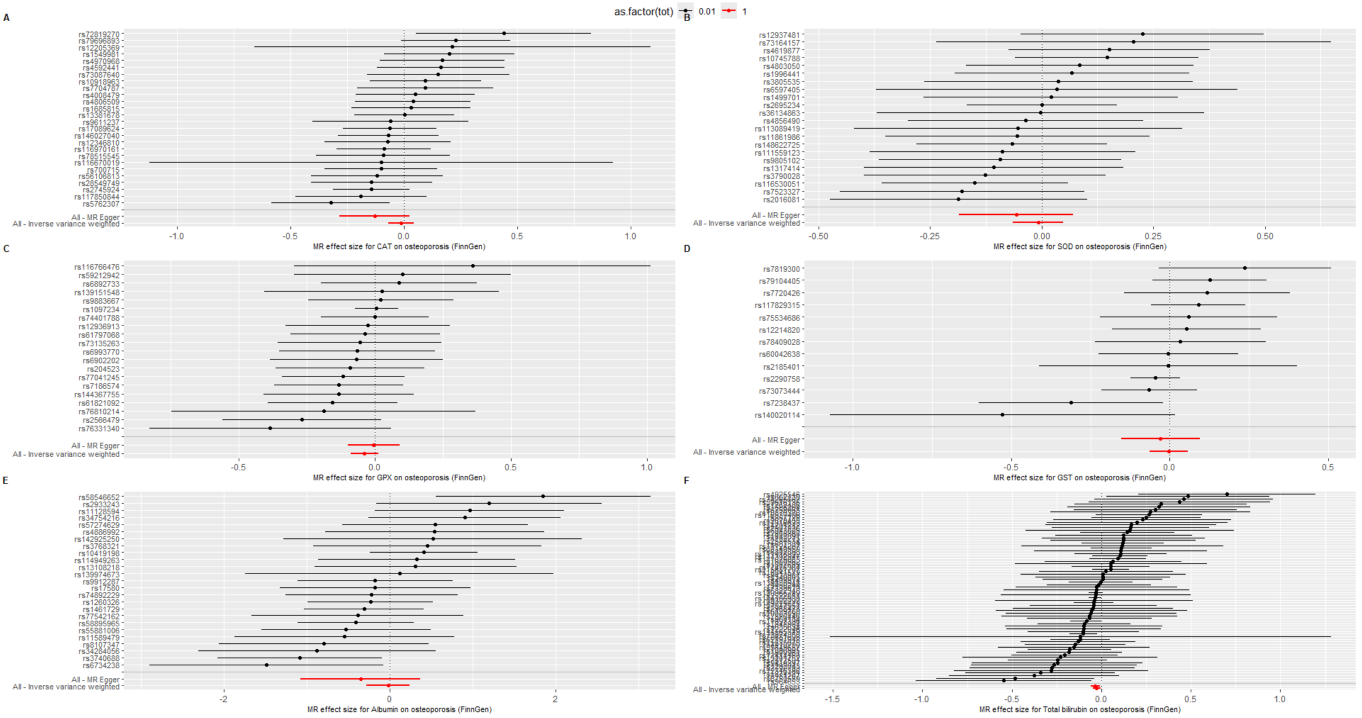

Figure S19. Forest plot:Endogenous Antioxidants on Osteoporosis(MRC-IEU)

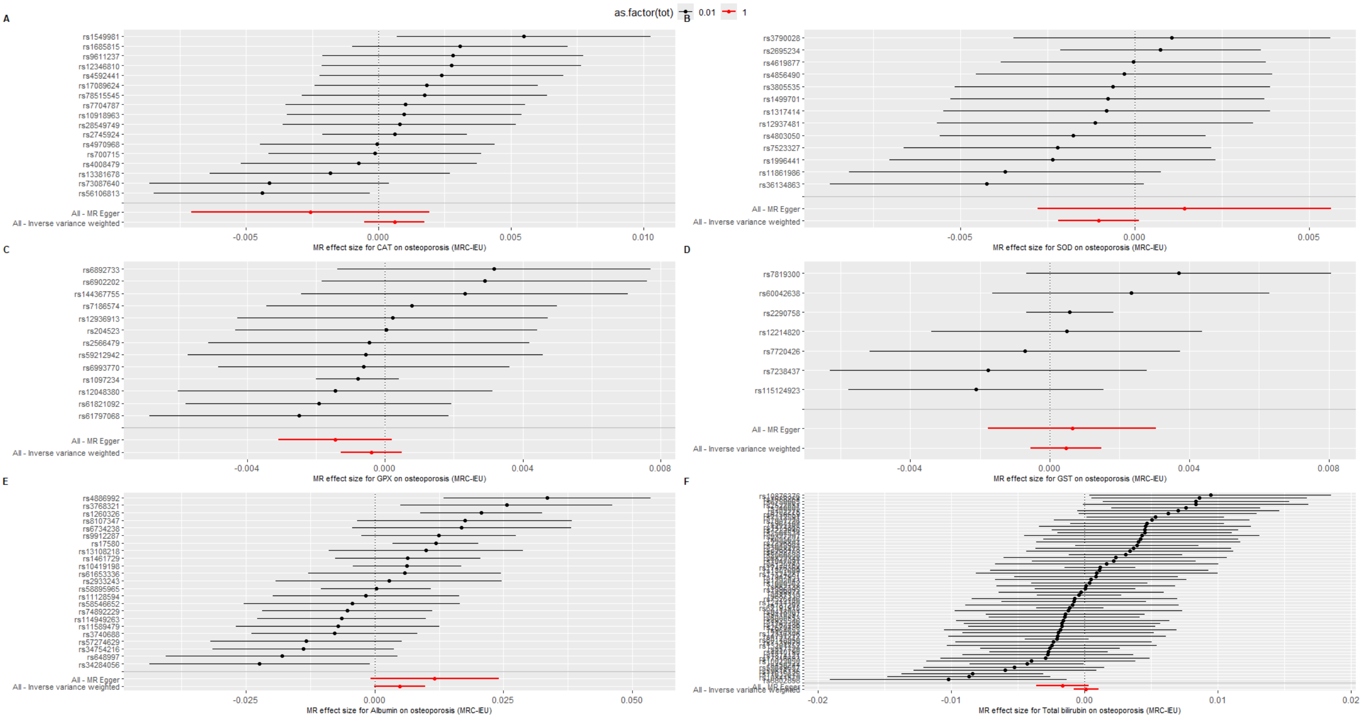

Figure S20. Forest plot:Endogenous Antioxidants on Osteoporosis(Neale Lab)

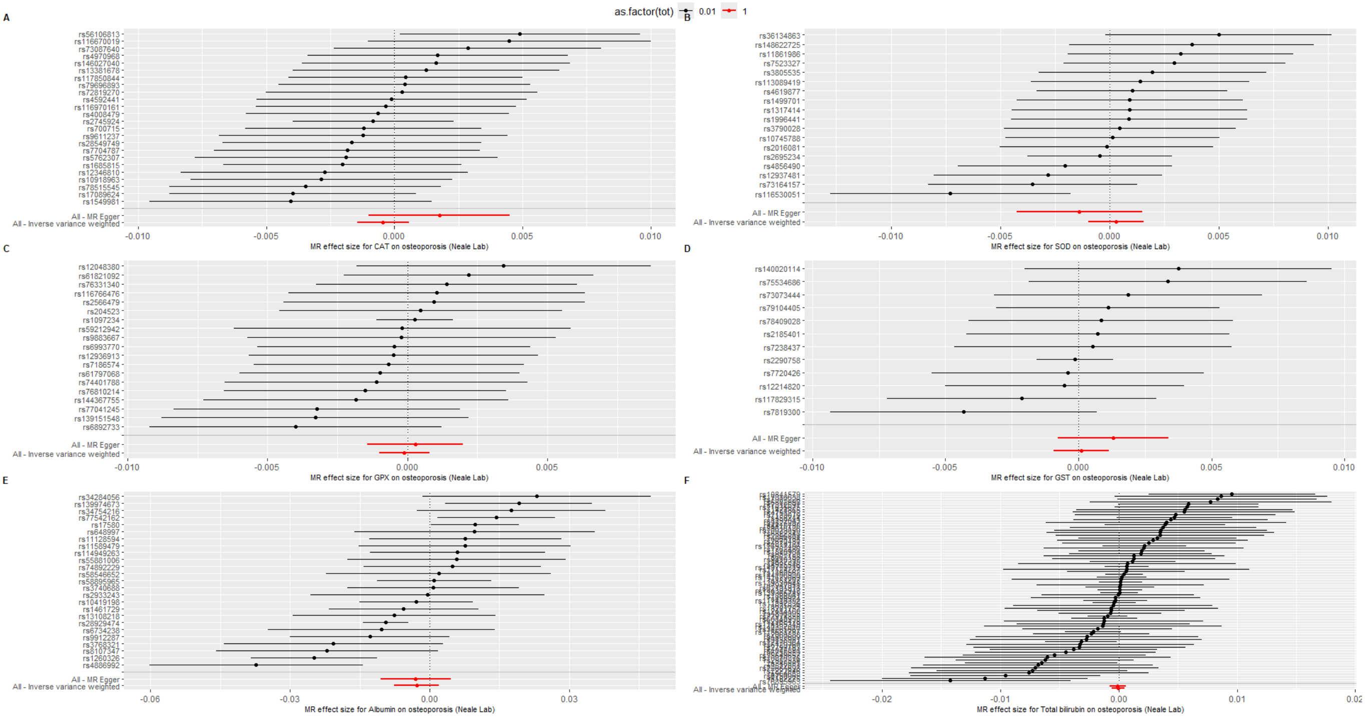

Figure S21. Forest plot:Osteoporosis on Endogenous Antioxidants (FinnGen)

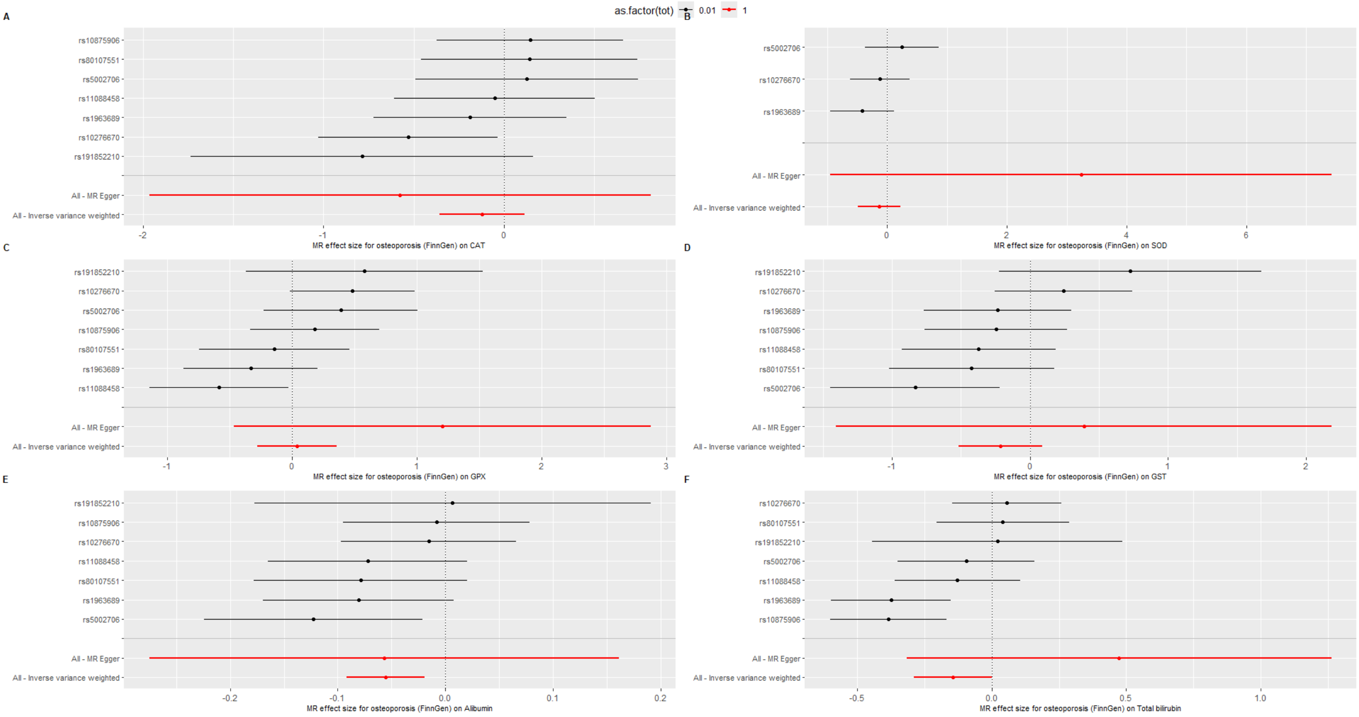

Figure S22. Forest plot:Osteoporosis on Endogenous Antioxidants (MRC-IEU)

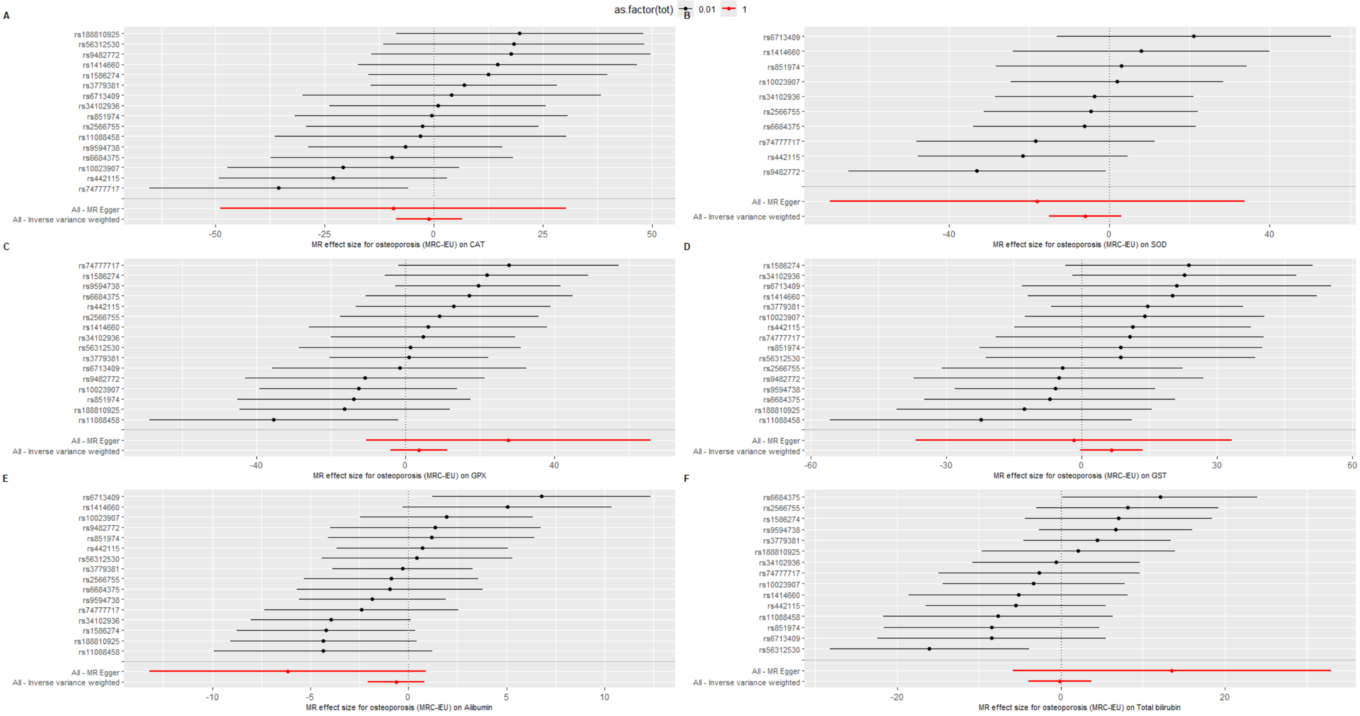

Figure S23. Forest plot-Osteoporosis on Endogenous Antioxidants (Neale Lab)

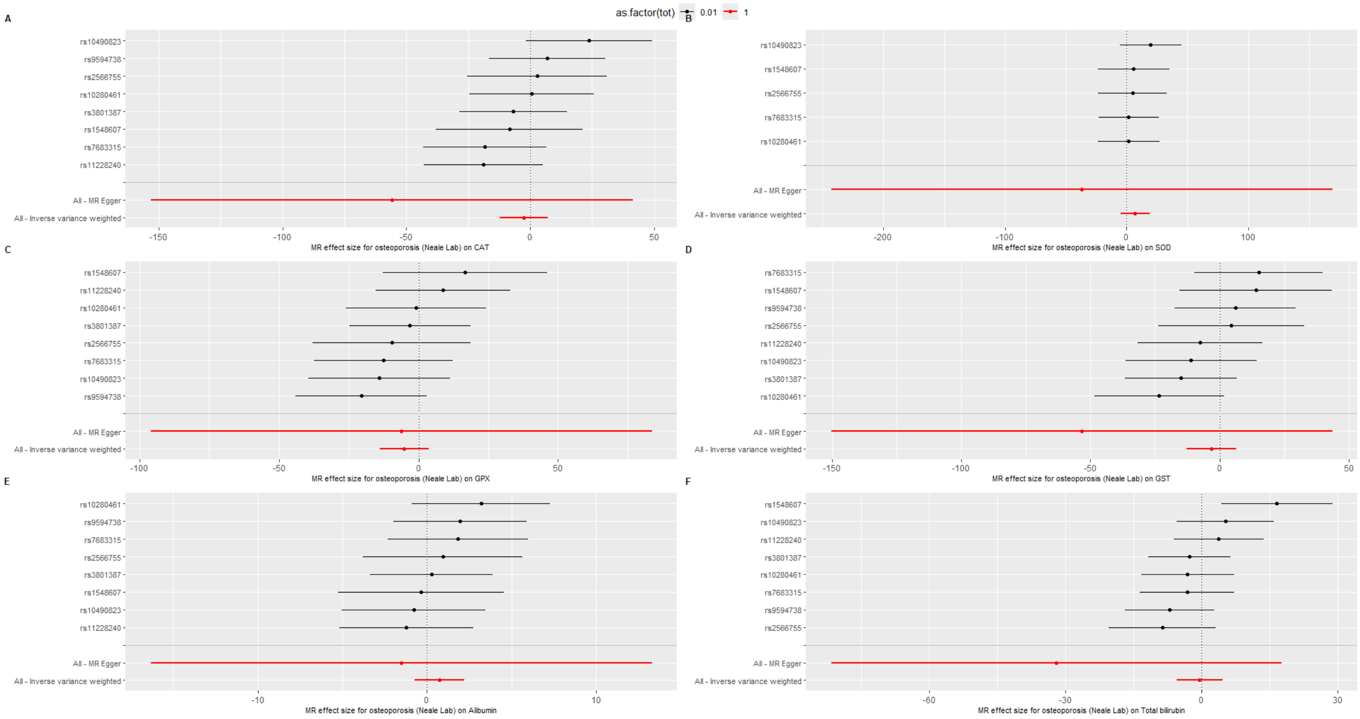

Figure S24. Forest plot:Exogenous Antioxidants on Osteoporosis(MRC-IEU)

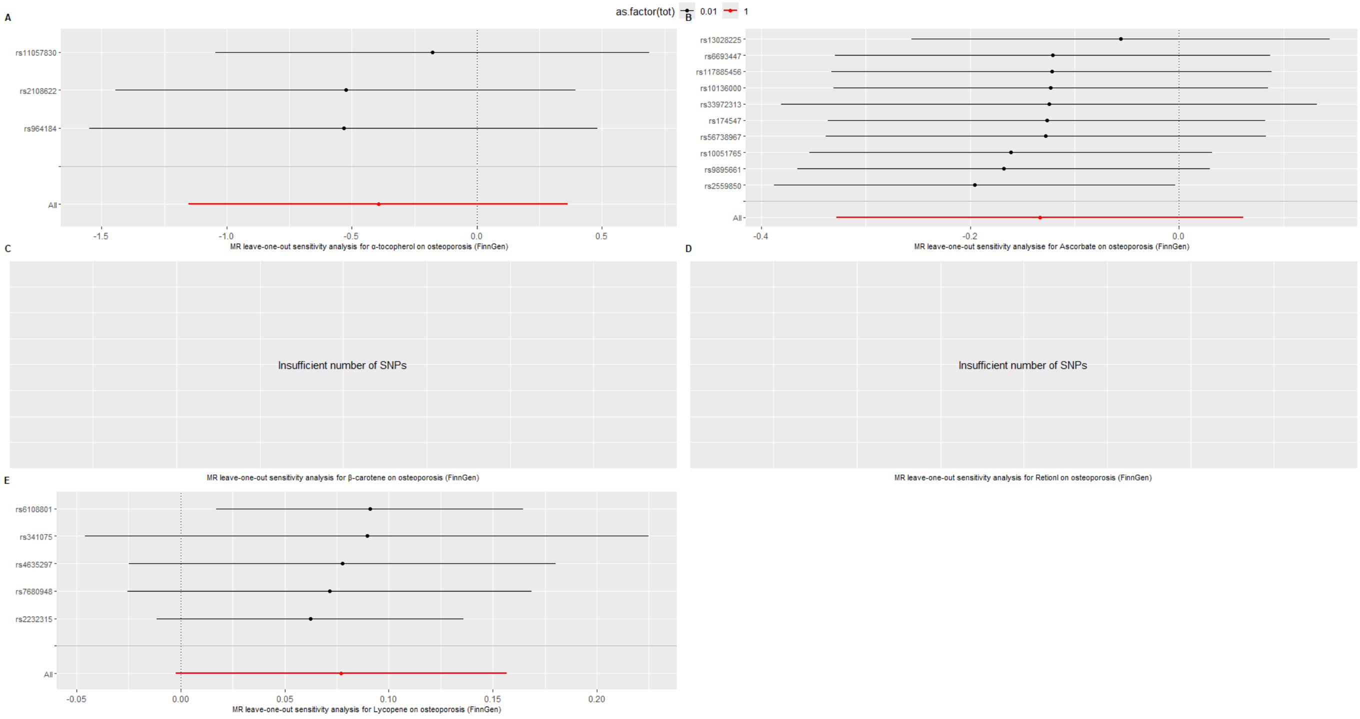

Figure S25. Leave-one-out plot:Exogenous Antioxidans on Osteoporosis(FinnGen)

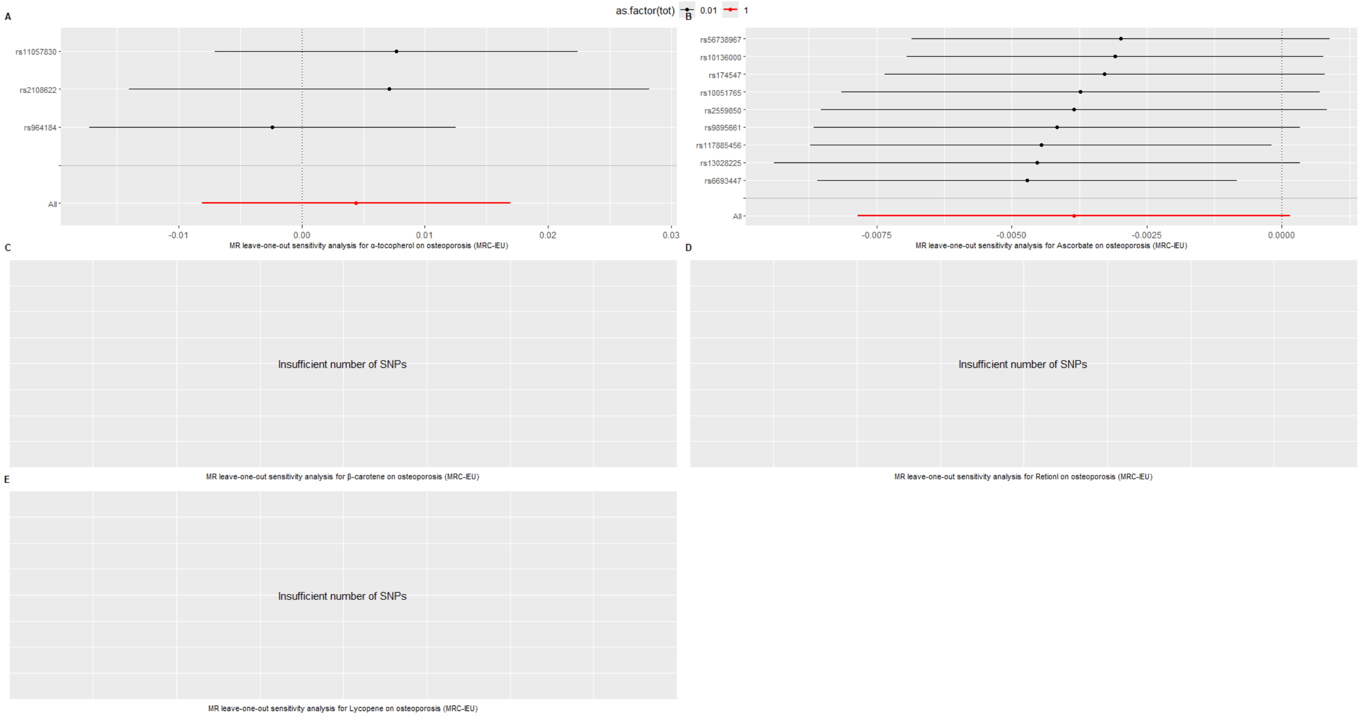

Figure S26. Leave-one-out plot:Exogenous Antioxidants on Osteoporosis(MRC-IEU)

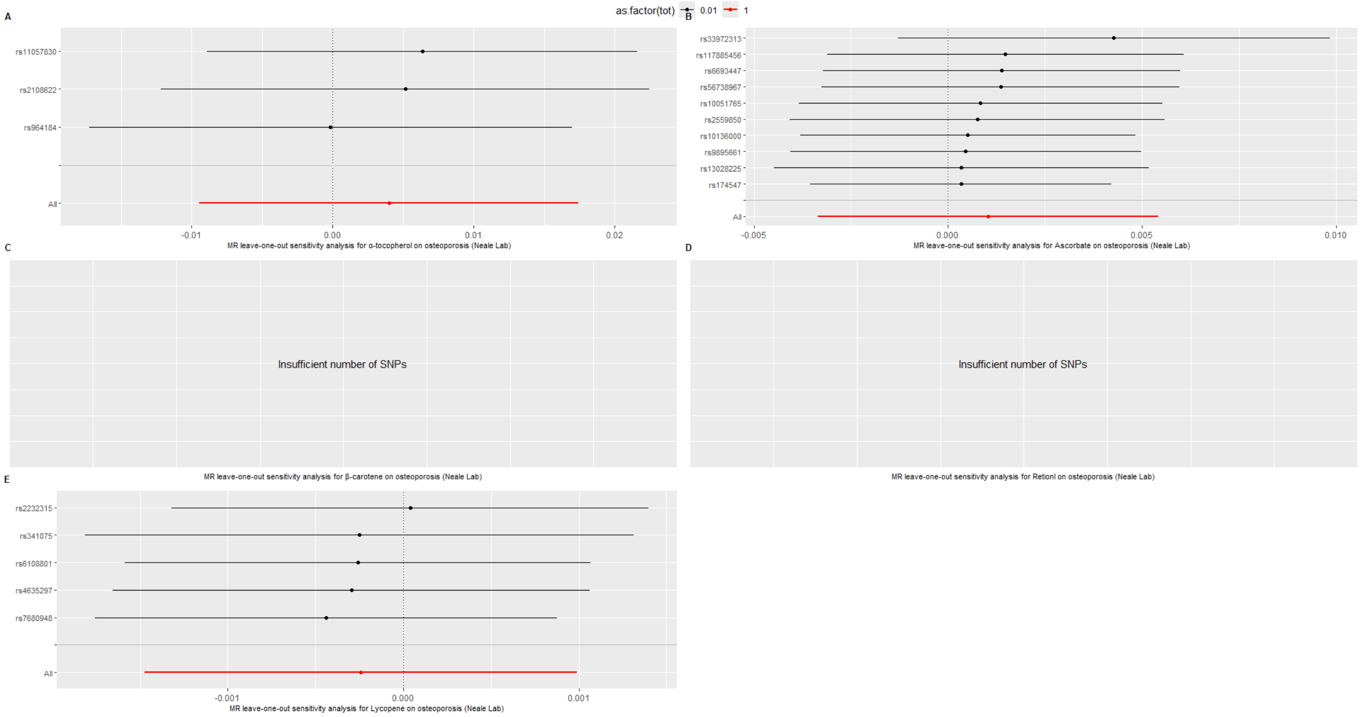

Figure S27. Leave-one-out plot:Exogenous Antioxidants on Osteoporosis(Neale Lab)

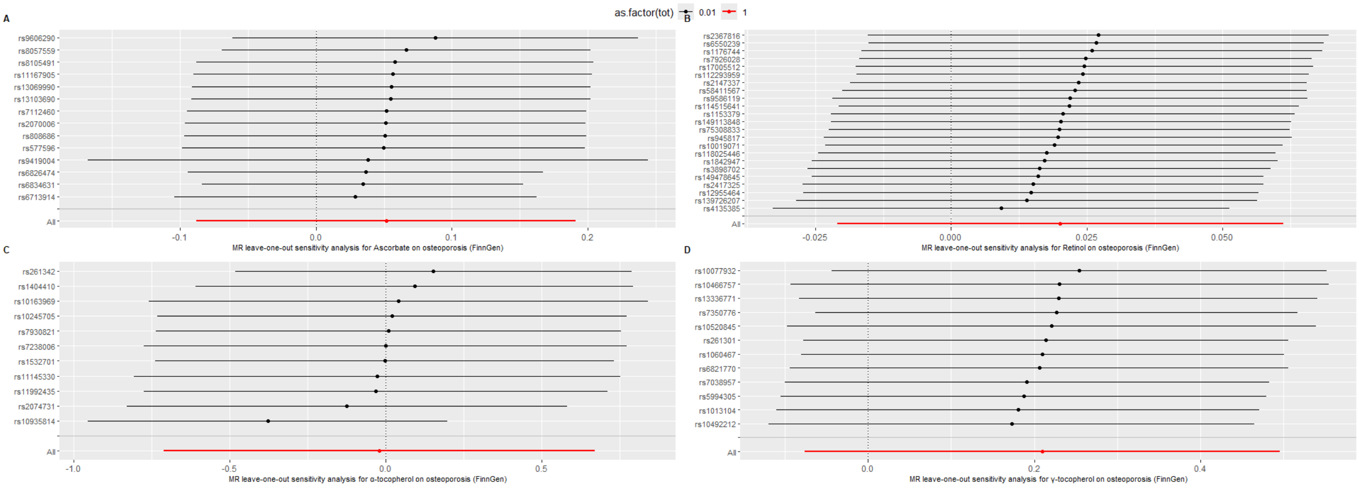

Figure S28. Leave-one-out plot:Exogenous Antioxidant Metabolites on Osteoporosis(FinnGen)

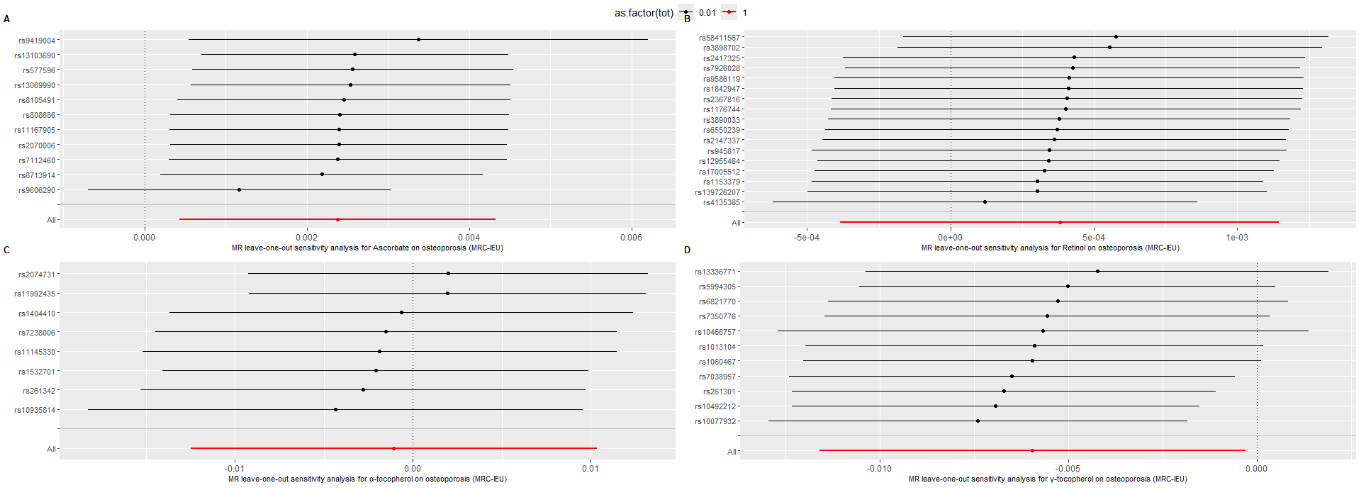

Figure S29. Leave-one-out plot:Exogenous Antioxidant Metabolites on Osteoporosis(MRC-IEU)

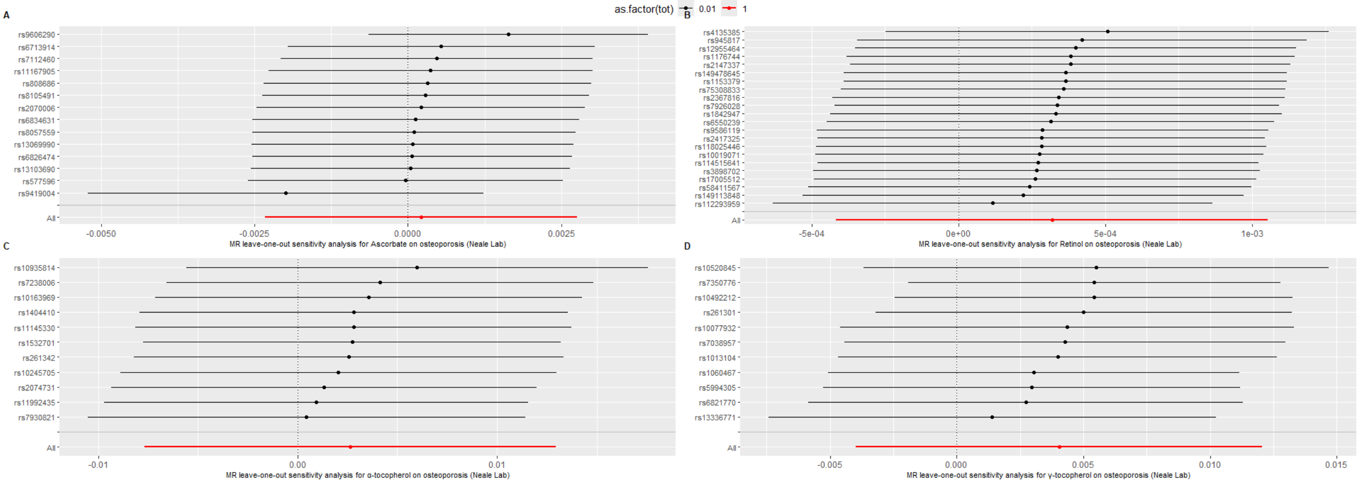

Figure S30. Leave-one-out plot:Exogenous Antioxidant Metabolites on Osteoporosis(Neale Lab)

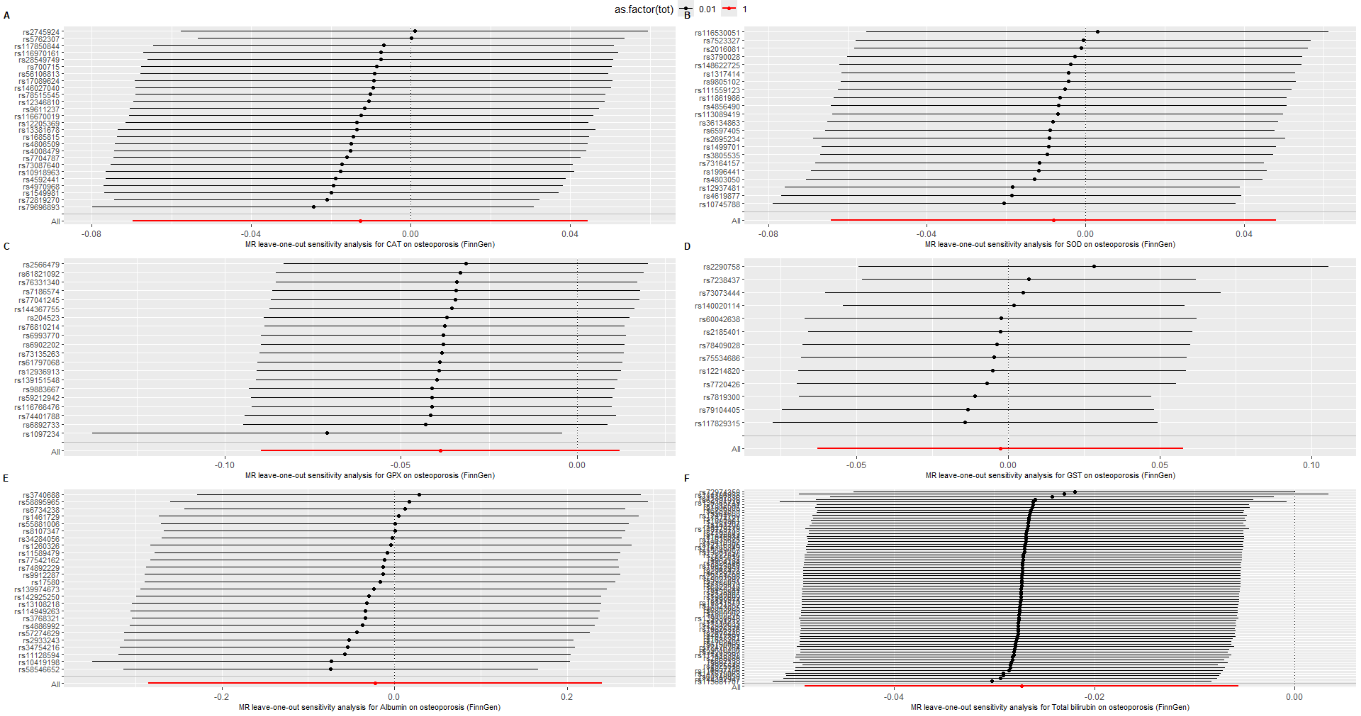

Figure S31. Leave-one-out plot:Endogenous Antioxidants on Osteoporosis(FinnGen)

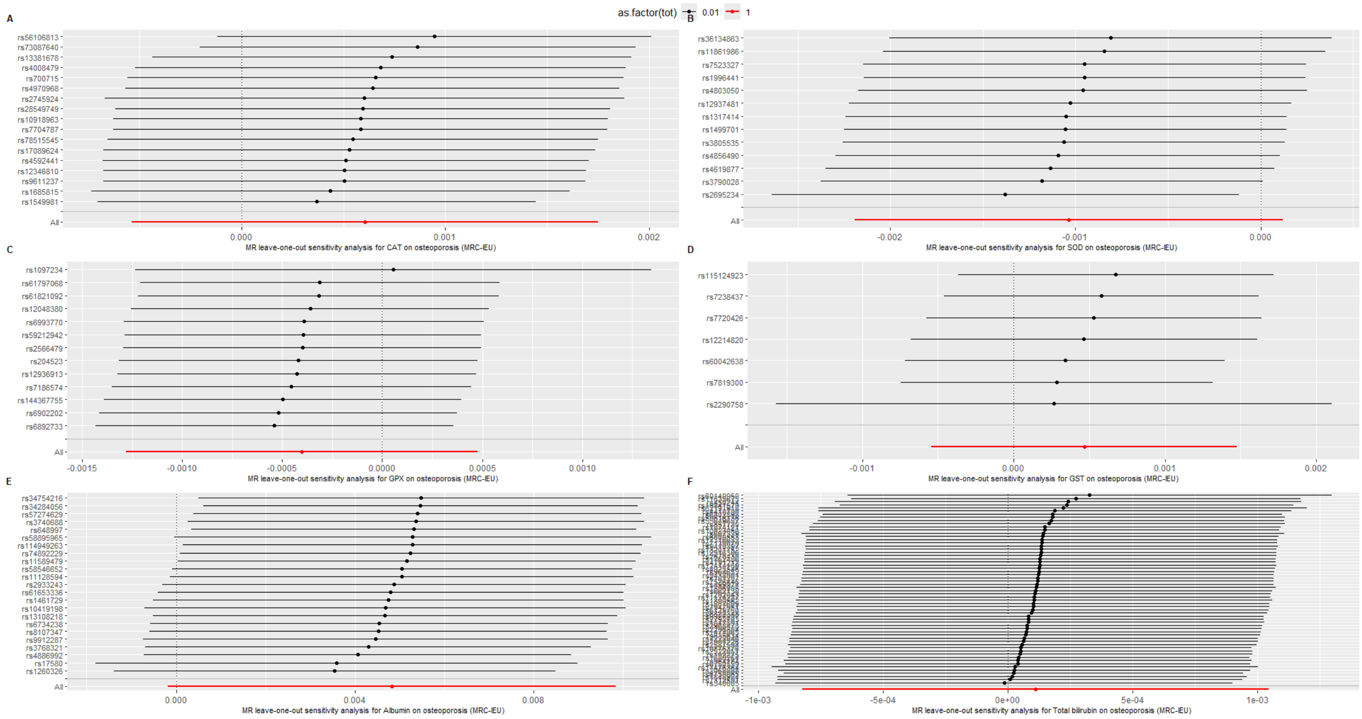

Figure S32. Leave-one-out plot:Endogenous Antioxidants on Osteoporosis(MRC-IEU)

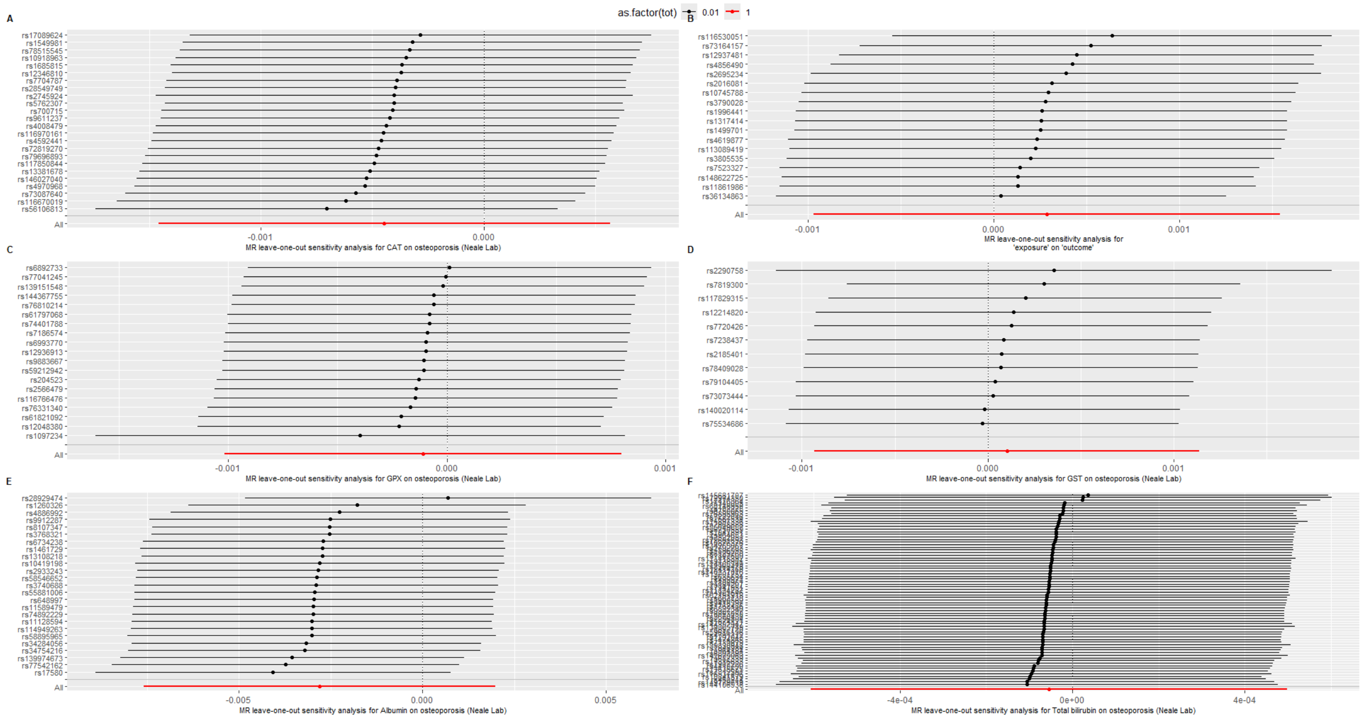

Figure S33. Leave-one-out plot:Endogenous Antioxidants on Osteoporosis(Neale Lab)

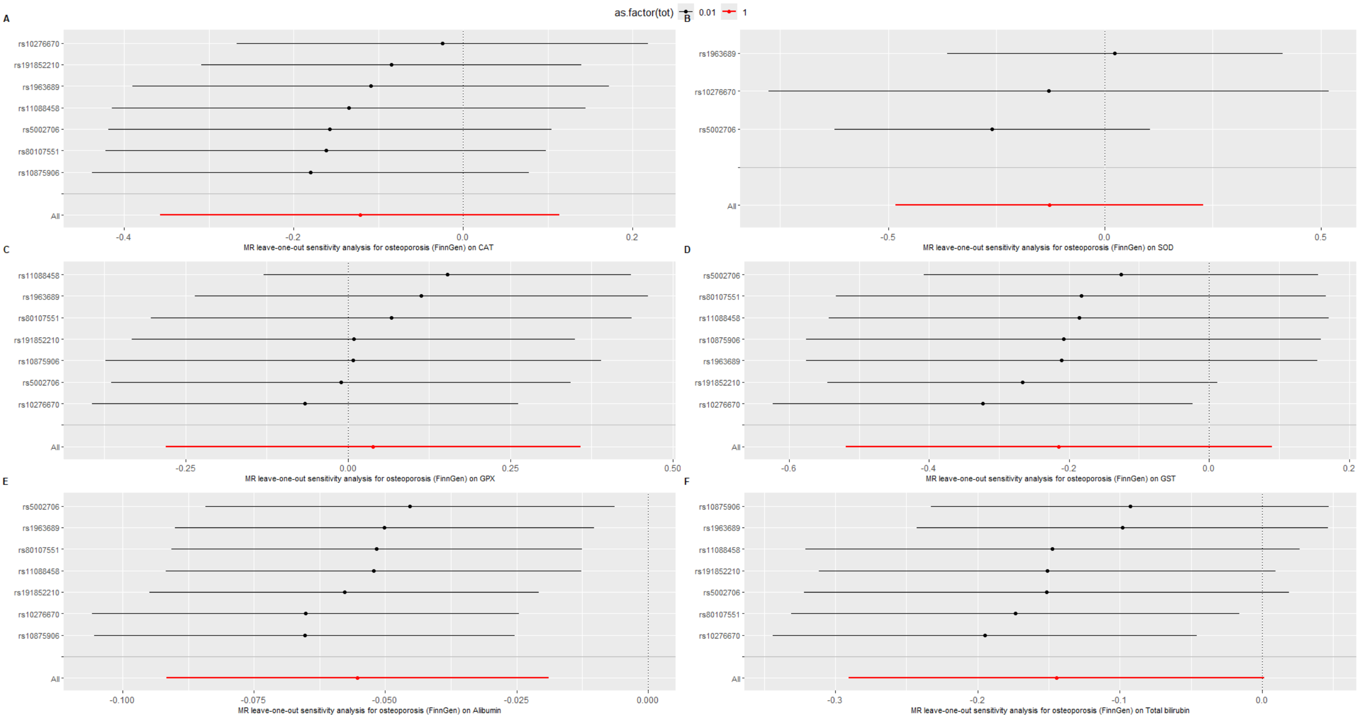

Figure S34. Leave-one-out plot:Osteoporosis on Endogenous Antioxidants (FinnGen)

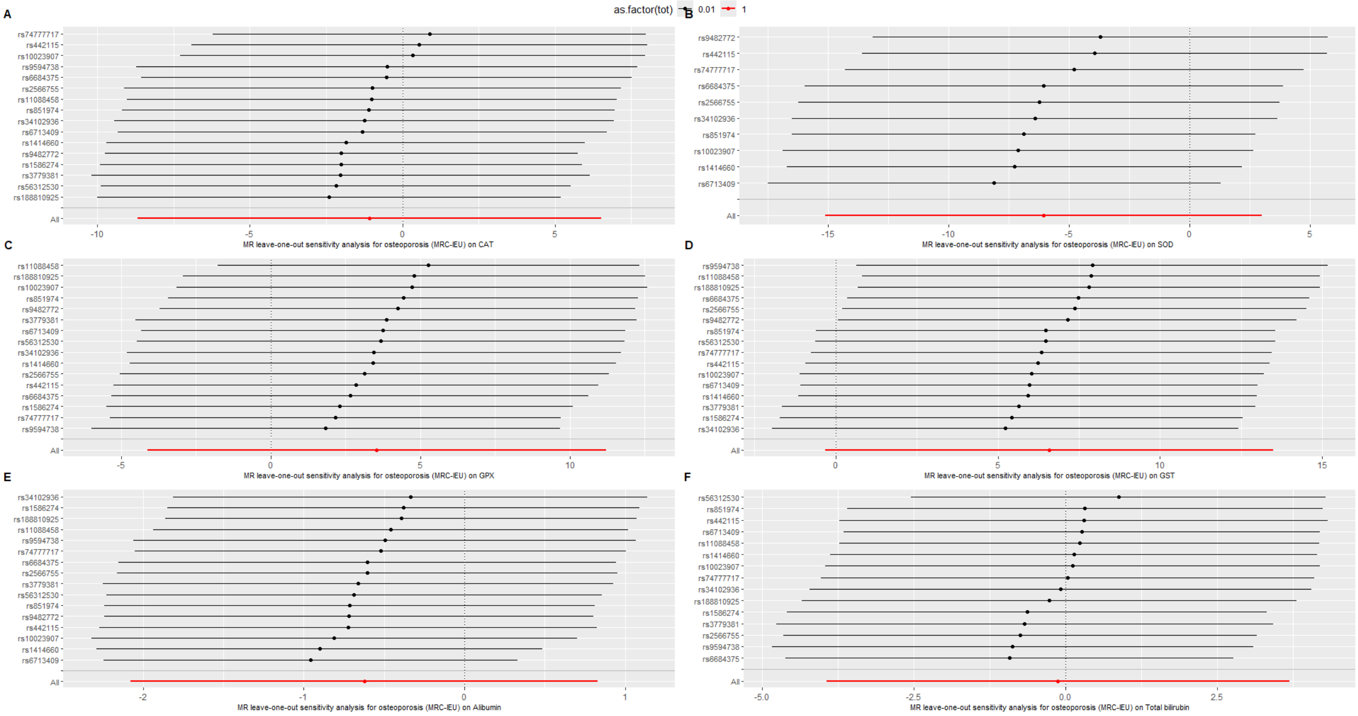

Figure S35. Leave-one-out plot:Osteoporosis on Endogenous Antioxidants (MRC-IEU)

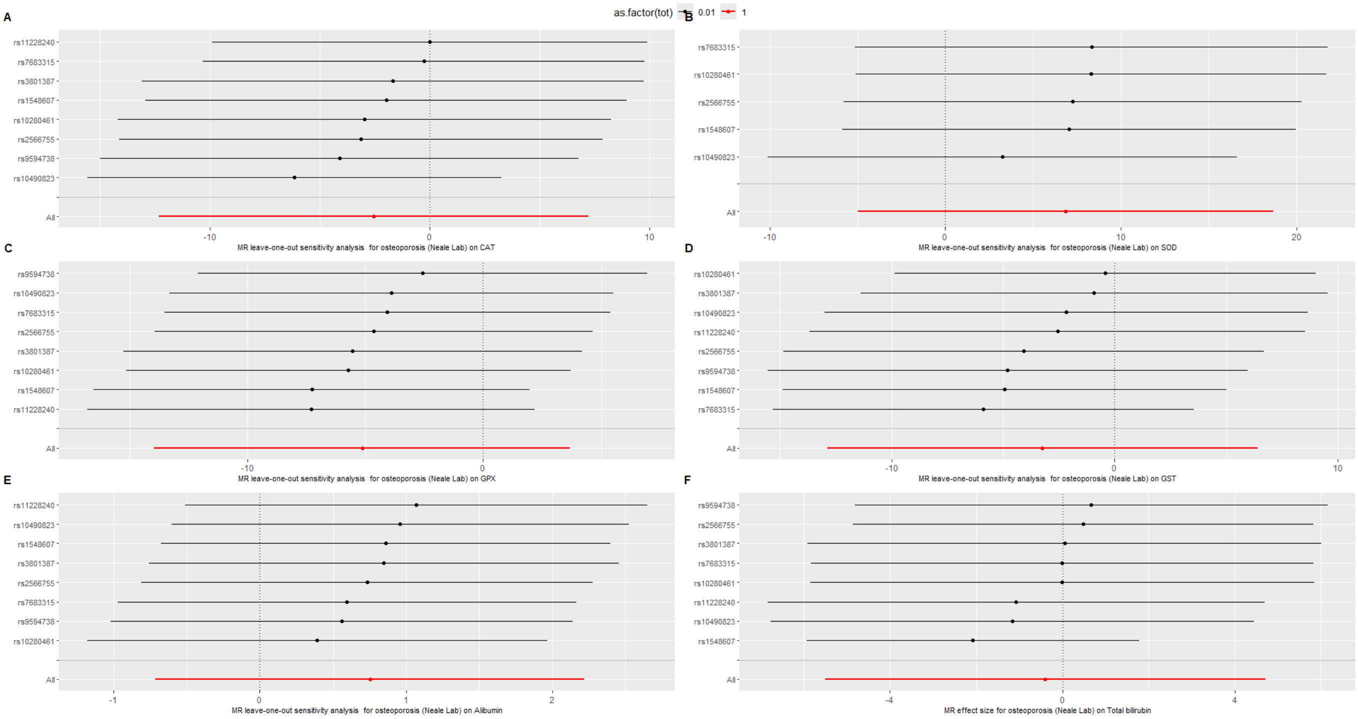

Figure S36. Leave-one-out plot-Osteoporosis on Endogenous Antioxidants (Neale Lab)
